# Unveiling the NEFH+ malignant cell subtype: Insights from single-cell RNA sequencing in prostate cancer progression and tumor microenvironment interactions

**DOI:** 10.3389/fimmu.2024.1517679

**Published:** 2024-12-20

**Authors:** Jie Wang, Fu Zhao, Qiang Zhang, Zhou Sun, Zhikai Xiahou, Changzhong Wang, Yan Liu, Zongze Yu

**Affiliations:** 1 Department of Urology, The Second People’s Hospital of Meishan City, Meishan, Sichuan, China; 2 Department of Urology, China-Japan Union Hospital of Jilin University, Changchun, Jilin, China; 3 The First Clinical Medical College of Shandong University of Traditional Chinese Medicine, Jinan, China; 4 China Institute of Sport and Health Science, Beijing Sport University, Beijing, China; 5 Department of Urology, The First People’s Hospital of Jiangxia District, Wuhan, Hubei, China; 6 Department of Urology, Xiangyang Central Hospital, Affiliated Hospital of Hubei University of Arts and Science, Xiangyang, Hubei, China

**Keywords:** multi-omics, single-cell RNA sequencing, prostate cancer, tumor heterogeneity, precision medicine, drug discovery

## Abstract

**Background:**

Prostate cancer (PCa) is a multifactorial and heterogeneous disease, ranking among the most prevalent malignancies in men. In 2020, there were 1,414,259 new cases of PCa worldwide, accounting for 7.3% of all malignant tumors. The incidence rate of PCa ranks third, following breast cancer and lung cancer. Patients diagnosed with high-grade PCa frequently present with existing or developing metastases, complicating their treatment and resulting in poorer prognoses, particularly for those with bone metastases. Utilizing single-cell RNA sequencing (scRNA-seq), we identified specific malignant cell subtypes that are closely linked to high-grade PCa. By investigating the mechanisms that govern interactions within the tumor microenvironment (TME), we aim to offer new theoretical insights that can enhance the prevention, diagnosis, and treatment of PCa, ultimately striving to improve patient outcomes and quality of life.

**Methods:**

Data on scRNA-seq was obtained from the GEO database. The gene ontology and gene set enrichment analysis were employed to analyze differential expression genes. Using inferCNV analysis to identify malignant epithelial cells. We subsequently employed Monocle, Cytotrace, and Slingshot packages to infer subtype differentiation trajectories. The cellular communication between malignant cell subtypes and other cells was predicted using the CellChat package. Furthermore, we employed pySCENIC to analyze and identify the regulatory networks of transcription factors (TFs) in malignant cell subtypes. The MDA PCa 2b and VCap cell lines were employed to validate the analysis results through cellular functional experiments. In addition, a risk scoring model was developed to assess the variation in clinical characteristics, prognosis, immune infiltration, immune checkpoint, and drug sensitivity.

**Results:**

A malignant cell subtype in PCa with high expression of *NEFH* was identified through scRNA-seq analysis. This subtype was situated at the differentiation terminal, exhibited a higher level of malignancy, and exhibited characteristics that were more prone to advanced tumor lesions. In addition, our research underscored the intricate interactions that exist within the TME, particularly the interaction between PTN secreted by this subtype and fibroblasts via the NCL receptor. This interaction may be closely associated with cancer-associated fibroblasts and tumor progression. Subsequently, we determined that the *NEFH*+ malignant cell subtype was significantly correlated with the TF IRX4. This TF is linked to a worse prognosis in PCa and may affect disease progression by regulating gene transcription. Our conclusions were additionally verified through cellular experiments. Furthermore, the prognostic model we developed demonstrated satisfactory predictive performance, with gene sets from the high NmRS group facilitating tumor progression and deterioration. The analysis of immune infiltration was instrumental in the development of clinical intervention strategies and patient prognosis.

**Conclusion:**

By examining the cellular heterogeneity of a unique *NEFH+* malignant cell subtype within the PCa microenvironment, we were able to disclose their reciprocal interaction with disease progression. This offers a novel viewpoint on the diagnosis and treatment of PCa.

## Introduction

Prostate cancer (PCa) is the second most common cancer worldwide and one of the leading causes of cancer-related death in men, according to the 2020 global cancer data released by the International Agency for Research on Cancer (1). Although most PCa patients are diagnosed with indolent or slow-progressing disease, approximately 15% of patients are diagnosed with high-risk cancer that can be life-threatening (2). Additionally, there are significant differences in the incidence and mortality rates of PCa among different racial and ethnic groups. The incidence rate among black patients is 70% higher than that among white patients, and the mortality rate is 2 to 4 times higher compared to other racial and ethnic groups (3). The treatment of PCa remains a challenging issue for clinicians and researchers, particularly in the case of metastatic disease. Bone metastasis is the most common site of metastasis in PCa and is a major cause of patient mortality. Approximately 1.7% to 11.9% of patients have bone metastasis at the time of initial diagnosis, with a median survival of less than 3 years and a 5-year survival rate of only 3%, severely impacting patients’ quality of life (4, 5).

Androgen deprivation therapy (ADT) is the mainstay of treatment for PCa, but although tumors often initially respond to ADT, the treatment effect is usually temporary and frequently leads to the development of resistance (6, 7). Data indicate that 10% to 20% of PCa progresses to castration-resistant prostate cancer (CRPC) within 5 years of diagnosis, with 84% of newly diagnosed CRPC patients experiencing metastasis (8). The median survival period for patients after the diagnosis of CRPC is approximately 14 months, which contributes to the increased mortality rate of PCa (7).

For patients with locally advanced high-grade disease who are not eligible for radical prostatectomy, external beam radiation therapy (EBRT) is commonly employed as the primary treatment modality (9). Although it is known that the addition of ADT to EBRT improves overall survival (OS) in patients with Gleason scores of 8-10 by approximately 1.5-fold compared to ADT alone (10, 11), the survival benefit from adding ADT is less pronounced for Gleason scores of 9-10, suggesting a lower sensitivity to ADT (12).

Currently, non-hormonal treatment options for advanced PCa mainly include chemotherapy and immunotherapy. Although studies have shown that the use of chemotherapy drugs may be beneficial in terms of survival, such as the efficacy of Docetaxel in symptomatic or rapidly progressing CRPC (13), the cytotoxic potential diminishes as androgen receptor-targeted therapy becomes the frontline treatment for resistant PCa. The rapid development of immunotherapy has brought revolutionary changes to the field of cancer treatment. However, the use of any single immunotherapy modality is unlikely to significantly alter the outcomes of PCa. Studies have shown that combining cancer vaccines or immune checkpoint inhibitors with other immunotherapeutic agents, hormonal therapies, radiation therapy, DNA damaging agents, or chemotherapy can enhance immune functionality and provide clinical benefits (14, 15). However, the limitations of immunotherapy are also quite apparent, including limited efficacy in the majority of patients, low response rates, and the potential for immune-related adverse effects, which are significant contributors to treatment failure in PCa. Furthermore, current treatment approaches have not fully addressed individual variability, resulting in a lack of broad applicability. Future research should focus on exploring novel immunotherapeutic strategies, optimizing combination therapies, and enhancing the study of patient biomarkers to improve efficacy and ensure a wider spectrum of beneficiaries (16).

PCa is a disease characterized by significant heterogeneity, and the mechanisms underlying its occurrence and progression are complex and dynamic. Previous research has highlighted the interaction between malignant epithelial cells and the tumor microenvironment (TME) as a key driver of PCa progression (17). Furthermore, we believe that cellular heterogeneity plays a critical role within the TME of PCa. Cellular heterogeneity reflects the diversity among different cell populations in the PCa microenvironment, which not only directly influences tumor growth and dissemination mechanisms but also establishes complex interactions among these cells. Investigating this heterogeneity can help elucidate the evolutionary pathways of PCa and deepen our understanding of its progression mechanisms, thereby providing essential insights for developing more targeted therapeutic strategies. In recent years, the development of single-cell sequencing technology has provided a new tool for studying the heterogeneity of tumor cells and the TME. By evaluating thousands of cells simultaneously, single-cell sequencing technology can reveal the complexity of intratumoral cells and provide new insights into the field of tumor biology, thereby improving the diagnosis and treatment of PCa and enhancing patient prognosis and survival rates. Therefore, we performed single-cell sequencing analysis on a PCa dataset from the GEO database, providing new perspectives for the diagnosis and treatment of PCa to improve patient prognosis and survival rates.

## Methods

### Acquisition and processing of single-cell derived data

The PCa data obtained through scRNA-seq was retrieved from the NCBI Gene Expression Omnibus (GEO) database (https://www.ncbi.nlm.nih.gov/geo/). The dataset used for single-cell analysis included 32 tumor and non-tumor samples from 18 PCa patients, with the GSE accession number GSE181294. Detailed information about the samples, including Tumor, Adj-normal, grade, Gleason, Grade Group, Path Stage, Margin, Age, etc., can be found in **Supplementary Table S1**. Bulk RNA-seq datasets and clinical data were obtained from the Cancer Genome Atlas (TCGA) (https://portal.gdc.cancer.gov/). The data used in this study was obtained from publicly available databases and therefore did not undergo ethical review.

The scRNA-seq data was imported into R software (version 4.2.0) and examined using the Seurat package (version 4.3.0) (18–21). We performed rigorous quality control on the data excluding the following cells: (1) 300 nFeature < 7,500; (2) 500 nCount < 100,000; (3) mitochondrial gene expression exceeding 25% of the total gene count within the cell; (4) red blood cell gene expression exceeding 5% of the total gene count within the cell. We kept 127,930 cells ultimately for more study. Our research did not call for ethical permission as we made use of publicly accessible database data.

Normalization and selection of the top 2000 highly variable genes (22–26) were performed on the filtered samples using the “NormalizeData” and “FindVariableFeatures” functions in the Seurat package. To correct for batch effects between datasets, principal component analysis was conducted using the Harmony R package (version 0.1.1) (27, 28). Cells were clustered using the FindClusters function with a resolution of 1.0 based on the top 30 principal components (PCs). The top 30 significant PCs were selected for uniform manifold approximation and projection (UMAP) dimensionality reduction and visualization of gene expression (29, 30).

### Single cells copy number variation evaluation

The scRNA-seq data was analyzed for CNVs using the inferCNV R program (version 1.6.0) available from the GitHub repository of the Broad Institute (https://github.com/broadinstitute/inferCNV). This software application facilitates the differentiation between malignant and healthy cells by examining the chromosomal positions and gene expression levels to ascertain CNVs (31, 32). Tumor-EPCs were identified as cells with high CNV scores.

### Cell type identification

We used Seurat’s “FindAllMarkers” function (33) to conduct a Wilcoxon rank-sum test with the goal of identifying differentially expressed genes (DEGs) among various cell clusters in order to examine the heterogeneity of PCa cells (34). Threshold = 0.25, min.pct = 0.25, and min.diff.pct = 0.25 were the parameters that were employed. Then, we used Seurat’s “DotPlot” and “featureplot” programs to show the expression patterns of the DEGs in each cluster. Manually reviewing the outcomes and consulting pertinent literature helped with the cell annotation process. Additionally, we regrouped these cells in order to investigate the heterogeneity of malignant cells in greater detail. We used marker identification to characterize each subtype based on the genes unique to that grouping.

### Enrichment analysis

The Gene Ontology (GO) (35, 36) and Gene Set Enrichment Analysis (GSEA) (37) tools were used to conduct enrichment studies of DEGs in distinct cell types. We performed a functional analysis of Kyoto Encyclopedia of Genes and Genomes (KEGG) utilizing the ClusterProfiler R package (version 4.6.2) (38–42).

One technique for gene set enrichment analysis is gene set variation analysis (GSVA). In order to determine enrichment scores for each gene set in each sample, it evaluates the variability of gene expression data and compares it to predefined gene sets.

### Pseudotime analysis and cell fate analysis

In order to examine the variations in development and differentiation amongst malignant subtypes in PCa, we created pseudotime trajectories of the subtypes using Monocle (v2.24.0), which showed the patterns of malignant cell differentiation (43). We utilized the CytoTRACE R package (version 0.3.3) (44) to evaluate cell fate, which enabled us to deduce the time course of cell differentiation. Next, we employed the Slingshot software (version 2.6.0) to deduce cell lineages as malignant subtypes differentiated (45). Each malignant subtype’s differentiation trajectory was deduced using the “getlineage” and “getCurves” tools. After that, a UMAP projection was created using these trajectories for visualization.

### Cell-cell communication analysis

We used the CellChat R package (version 1.6.1) (46) to compute regulatory networks based on ligand-receptor levels and infer complicated cell-to-cell interactions, as well as analyze the intercellular communication network within the TME. The “Identify Communication Patterns” function was used to estimate the number of communication patterns, and the “netVisualDiffInteraction” function was used to show the variation in communication strength between cells. The *P*-value, or significance threshold of 0.05, was used.

### Gene regulatory network construction

To analyze the scRNA-seq data and reconstruct gene regulatory networks, we used Python (v3.7) and the pySCENIC package (version 0.10.0) uncovering key gene regulatory mechanisms. To evaluate TFs enrichment and regulatory factor activity, we created an AUCell matrix for this investigation.

### Construction and validation of risk model

To construct a risk model, we obtained PCa-related data from the TCGA database (https://portal.gdc.cancer.gov/). Initially, we performed univariate Cox regression analysis to screen for potential prognostic-related genes (47–52). To account for multicollinearity among these genes, we further employed the least absolute shrinkage and selection operator (LASSO) regression (53–57) using the glmnet package (version 4.1-6). We assigned weights to each gene based on its expression levels and the coefficients obtained from the multivariable Cox regression analysis. This allowed us to construct a risk score formula: Risk score = ∑_i^n (Xi × Yi) (X: coefficient, Y: gene expression level). Next, using the “surv_cutpoint” function, we determined the optimal cutoff value to divide patients into high and low-risk groups based on the risk scores. The predictive accuracy of the model was assessed using ROC curve analysis (58). To observe the prognosis of patients in different groups, Kaplan-Meier survival curves were used to evaluate the survival differences between different risk groups (58, 59). Survival analysis was performed using the Survive package (version 3.3.1) and survminer package (version 0.4.9). To validate the predictive capabilities of the model based on *NEFH*+ malignant cell scores, we employed the “Survival” and “Time ROC” R packages to generate ROC curves for 1-year, 3-year, and 5-year survival rates, and calculated the area under curve (AUC) value of the ROC curve (60–65). Survival analysis and time-dependent ROC analysis were used for model validation. The distribution of risk score scores, scatter plots of survival status, and heatmaps were utilized to assess the model.

### TME immunoassay

For the evaluation of the TME, we employed the ESTIMATE R package (version 1.0.13) to estimate the stromal score, immune score, and ESTIMATE score in PCa tissues (66). To analyze RNA-Seq data and determine the relative proportion of infiltrating immune cells, we utilized the Cell Type Identification for Estimating Relative Subtypes of RNA Transcripts (CIBERSORT R package, version 0.1.0) algorithm (67, 68), which provides insights into 22 different immune cell types. Additionally, to quantify immune cell infiltration in each sample, we employed the xCell package to assess the enrichment of immune cells in PCa samples. Furthermore, we investigated the correlation between risk scores and immunomodulatory genes, particularly immune checkpoints. To evaluate the response to tumor immune therapy, we utilized the Tumor Immune Dysfunction and Exclusion (TIDE) tool (http://tide.dfci.harvard.edu).

### Mutation analysis

Using the TCGAbiollinks and maftools R package, the somatic mutation data of PCa were obtained from the TCGA database. The PCa expression data and TMB file were imported into the R package. The correlation between DEGs associated with PCa and tumor mutation burden was computed. Based on the correlation results, waterfall plots were generated to depict the high-risk and low-risk groups.

### Drug sensitivity analysis

With the pRRophetic R package (version 0.5), we employed the GDSC database (https://www.cancerrxgene.org/), the most comprehensive pharmacogenomics database, to predict the treatment response of each tumor sample (69, 70). The IC50 values for each medication were determined using regression, and the accuracy of both regression and prediction was assessed using 10-fold cross-validation with the GDSC training set. All settings were set to their default values, including the “combat” option for removing batch effects and the mean value for duplication gene expression (71–73).

### Cell culture

The MDA PCa 2b and VCap cell lines were acquired from ATCC and cultured in specific growth media. MDA PCa 2b cells were cultured in F12K medium, while VCap cells were cultured in DMEM medium. Both media were supplemented with 10% fetal bovine serum from Gibco BRL, USA, and 1% penicillin/streptomycin. The cells were incubated under standardized conditions, including a constant temperature of 37°C, a carbon dioxide concentration of 5%, and a humidity level of 95%.

### SiRNA transfection

IRX4 knockdown was achieved by employing short interfering RNA constructs obtained. The transfection process followed the prescribed guidelines supplied by Lipofectamine 3000 RNAiMAX (Invitrogen, USA). The cells were introduced to a six-well plate when they reached a 50% coverage and subsequently underwent transfection using negative controls (si-NC) as well as knockdown constructs (Si-IRX4-1 and Si-IRX4-2). The transfection process utilized Lipofectamine 3000 RNAiMAX (Invitrogen, USA) for each instance. The siRNA sequences are recorded in **Supplementary Table S2** in the **Supplementary Materials**.

### Cell viability assay

The CCK-8 assay was used to evaluate the cellular vitality of MDA PCa 2b and VCap cells after transfection. The cell suspensions were added to a 96-well plate with a density of 5×10³ cells per well and incubated for 24 hours. After adding 10μL of CCK-8 labeling reagent (A311-01, Vazyme) to each well, the plate was placed in a light-protected environment and incubated at 37°C for 2 hours. Cell viability was assessed by quantifying the absorbance at 450nm using an enzyme-linked immunosorbent assay (ELISA) reader on days 1, 2, 3, and 4. The mean optical density (OD) values were computed and depicted on a line graph.

### Cell proliferation assay with 5-Ethynyl-2’-deoxyuridine

MDA PCa 2b and VCap cells that had been genetically modified were placed in a 6-well plate at a concentration of 5×10³ cells per well and let to grow overnight. Afterwards, a solution of EdU with a concentration that is twice as strong as the original was prepared by combining 10 mM EdU with a medium that does not contain serum. The solution was introduced into the cell culture and permitted to incubate at a temperature of 37°C for a duration of 2 hours. After the incubation period, the liquid containing the cells was removed, and the cells were carefully rinsed with PBS. Subsequently, the cells were treated with a 4% paraformaldehyde solution for a duration of 30 minutes to ensure fixation. Afterwards, a glycine solution with a concentration of 2 mg/mL and 0.5% Triton X-100 was administered for a duration of 15 minutes. Subsequently, the cells were incubated at room temperature with a solution containing 1 ml of 1X Apollo and 1 ml of 1X Hoechst 33342 for a duration of 30 minutes. Fluorescence microscopy was used to measure and analyze cell proliferation.

### Wound healing assay

The cells were transfected, then grown to a cell density of 95% by seeding them onto 6-well plates. Then, using a sterile 200 μL pipette tip, a careful linear scratch was made over the cell layer in the culture wells. After that, PBS was used to gently wash the wells. After rinsing, the cell culture was let to continue and the culture medium was changed. Photography was used to record the scratches at the original time point (0 hours) and 48 hours later. Measurements were made of the scratches’ width for further examination.

### Transwell assay

To prepare for the experiment, the cells were subjected to a 24-hour serum-free medium starvation. Subsequently, cell suspensions were added to the upper chamber, which contained Costar, after the addition of matrix gel (BD Biosciences, USA). In the lower chamber, serum-containing medium was added. The cells were then incubated in a cell culture incubator for 48 hours. After the incubation period, the cells were fixed with 4% paraformaldehyde and stained with crystal violet to evaluate their invasive capacity.

### Statistical analysis

We performed statistical analysis using R software and Python software to analyze the database data. All *p*-values reported in this study are two-tailed, with values less than 0.05 considered statistically significant. *P*-values below 0.001 were considered highly significant, while those below 0.0001 were regarded as extremely significant.

## Results

### Single cell landscape of PCa

**Figure 1:**
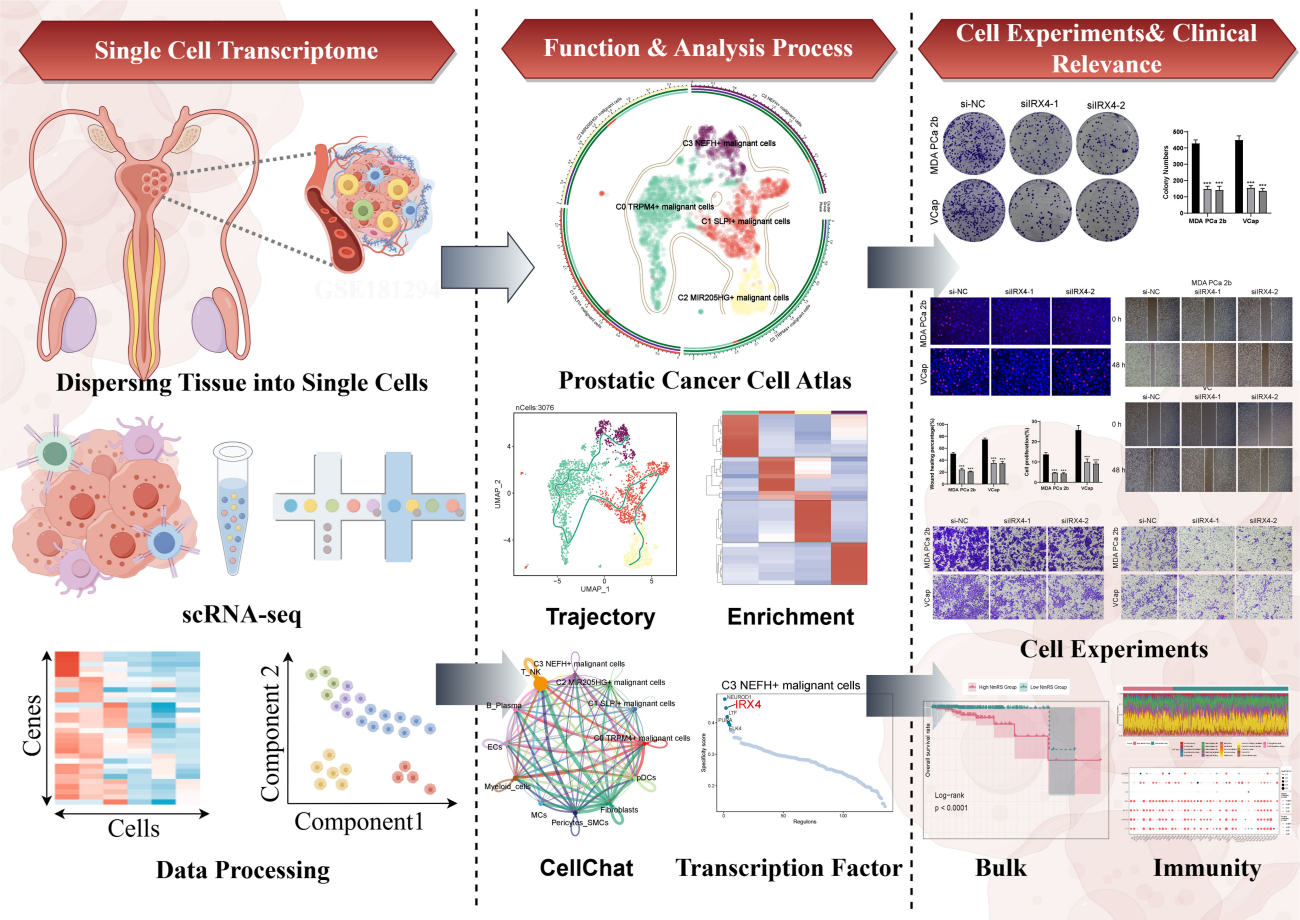
Graphical Abstract. The analysis workflow for this research. We performed single-cell sequencing analysis on the GSE181294 dataset and identified a distinct C3 *NEFH+* malignant cells subtype. Through pseudotime analysis, enrichment analysis, cell communication, and transcription factor regulation analysis, we revealed the significance of this subtype and confirmed the important role of the key TF (IRX4) through *in vitro* experiments. Prognostic and immune analyses provided guidance for clinical intervention and treatment.

**Figure 2:**
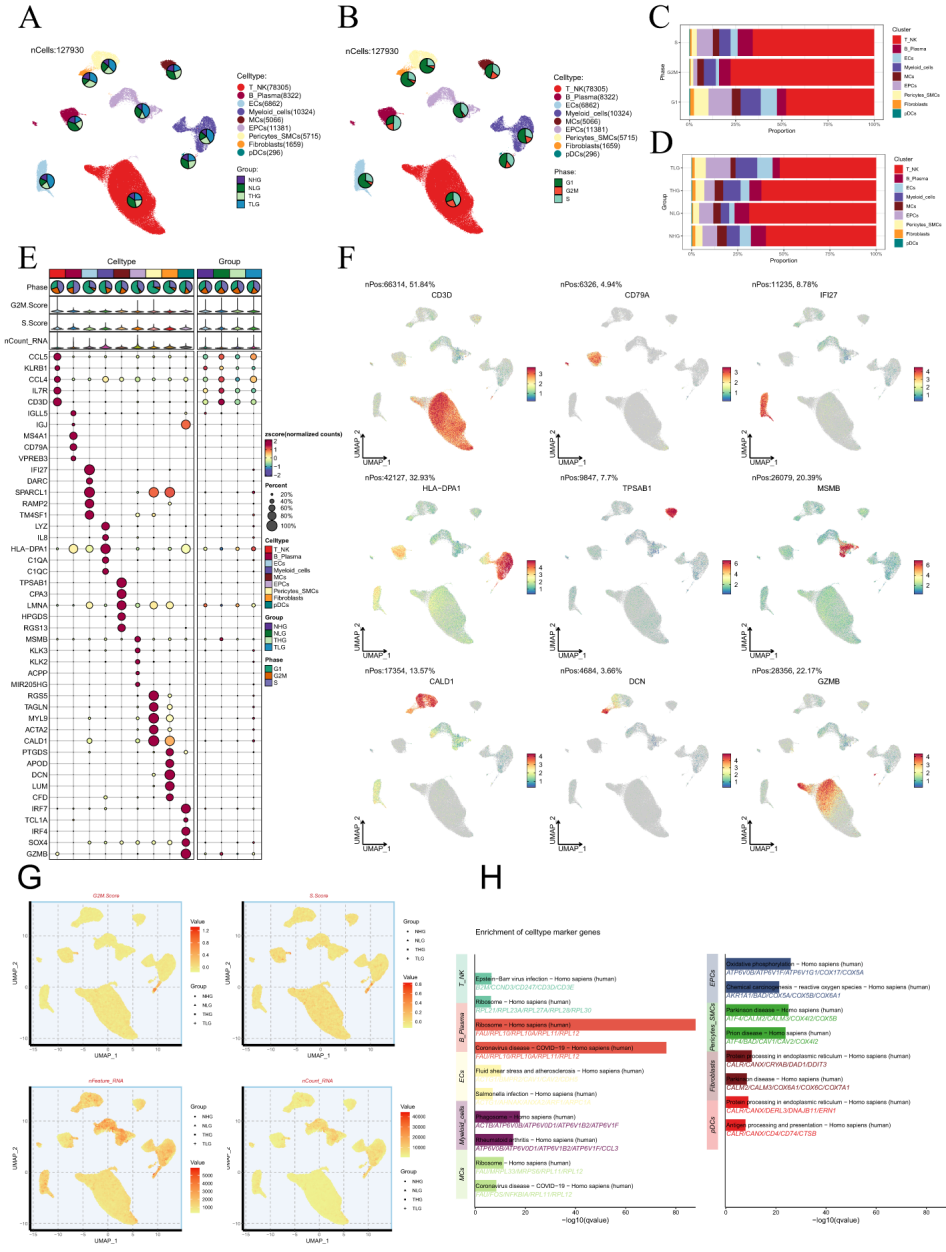
Single-cell analysis in PCa. **(A, B)** The UMAP plots depicted the single-cell lineage revealed in this work and labeled cell types using established marker genes (T-NK cells, B-plasma cells, ECs, myeloid-cells, MCs, EPCs, pericytes-SMCs, fibroblasts, and pDCs). The pie charts depicted the allocation of individual cell types across various Group and Phase classifications. **(C, D)** The stacked bar graphs illustrated the relative amounts and distributions of nine cell types in various tissue sources and stages of the cell cycle. **(E)** The bubble plot depicted the levels of gene expression for the five most significantly different genes across the nine cell types of PCa. The size of each bubble was proportional to the percentage of gene expression, while the color indicated data normalization. **(F)** The UMAP plots were used to show the differential gene expression in the nine cell types. **(G)** The UMAP plots displayed the G2/M scores, S scores, nFeature-RNA, and nCount-RNA of the nine cell types. **(H)** Conducting enrichment analysis to identify variations in biological processes across distinct cell types in PCa.

We conducted an extensive analysis of the obtained dataset to unveil the intricate single-cell landscape within the PCa microenvironment. Our workflow was illustrated in **Figure 1**. Using the known typical cell type marker genes, 127,930 high-quality cells were labeled. By using dimensionality reduction clustering with UMAP plot, 9 cell types were obtained: T-NK cells, B-plasma cells, endothelial cells (ECs), myeloid-cells, mast cells (MCs), epithelial cells (EPCs), pericytes-smooth muscle cells (pericytes-SMCs), fibroblasts, and plasmacytoid dendritic cells (pDCs). The proportions of different tissue types (Adjacent-Normal high-grade (NHG), Adjacent-Normal low-grade (NLG), Tumor high-grade (THG, Gleason 8–10), Tumor low-grade (TLG, Gleason 6 and 7) and cell cycle phases (G1, G2/M, S) in various cell types were visualized using pie charts (**Figures 2A, B**).

Subsequently, we analyzed the proportion of cell types in different phases (**Figures 2C, D**). We found that EPCs, pericytes-SMCs, fibroblasts, ECs, and myeloid-cells were mainly derived from tumor cells. The results of cell cycle study showed that T-NK cells occupied a larger proportion in G2/M and S phases. Then, we described the expression level and distribution of typical marker genes related to cell subtypes in cells (**Figures 2E, F**). By visualizing the analysis of G2/M.score, S.score, nFeature-RNA and nCount-RNA of all cells, the differences among cell types were further clarified (**Figure 2G**). The results of enrichment analysis showed that the marker genes of EPCs were mainly enriched in oxidative phosphorylation pathway (**Figure 2H**). It is worth noting that PCa is mainly transformed from glandular epithelial cells of prostate (74), and oxidative phosphorylation is considered as a tumor-related metabolic marker (75), which confirms that our findings are consistent with the recognized biological functions related to PCa.

### Visualization of malignant cell subtypes in PCa

**Figure 3:**
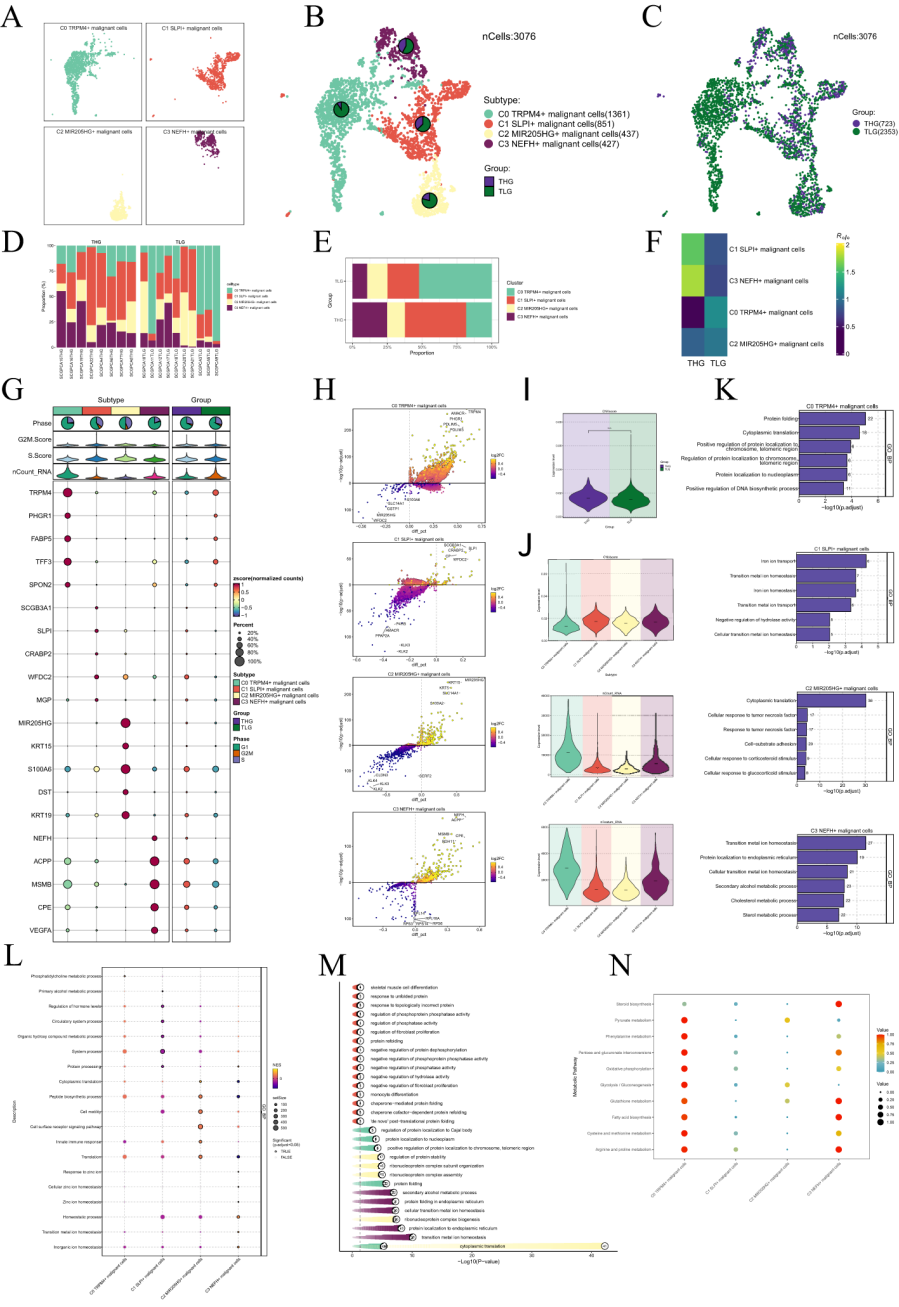
Visualization of malignant cell subtypes in PCa. **(A)** Malignant cells were annotated based on known specific marker genes (represented by color): C0 *TRPM4+* malignant cells, C1 *SLPI*+ malignant cells, C2 *MIR205HG+* malignant cells, and C3 *NEFH+* malignant cells. The UMAP plot was used to visualize the distribution of these four malignant cell subtypes. **(B)** Pie charts displayed the distribution of each malignant cell subtype in different tissue types based on the UMAP plot. The tissue types were categorized as THG and TLG. **(C)** The UMAP plot illustrated the distribution of malignant cells across different tissue types. **(D, E)** Bar graphs illustrated the relative proportions of the four malignant cell subtypes across various samples and tissue types. **(F)** The Ro/e score was used to evaluate the tissue preference of each subtype of malignant cells. **(G)** Bubble plots depicted the mean expression levels of the top five genes that were differently expressed in each malignant cell subtype. The size of each bubble was proportional to the percentage of gene expression, while the color indicated data normalization. **(H)** The volcano plots displayed the expression levels of significantly upregulated and downregulated genes in the four malignant cell subtypes. **(I–J)** The violin plots illustrated the levels of CNVscore, nFeature_RNA, and nCount_RNA in different tissue types and malignant cell subtypes. **(K–M)** Enrichment analysis results of biological processes in the malignant cell subtypes were presented. **(N)** Bubble plots visually represented the metabolic pathways in several malignant cell subtypes.

Given the profound importance of malignant cells in TME, our subsequent objective is to characterize these cells in the microenvironment of PCa. Studies indicated that a high CNV score typically signified significant copy number alterations within cells, which might have been associated with malignant transformation. CNV scoring could be utilized to identify tumor heterogeneity and reveal the presence of different subpopulations within the tumor microenvironment, aiding in the understanding of tumor development and resistance mechanisms (76, 77). To detect aberrant amplification or deletion of chromosome copy number in EPCs, we initially employed inferCNV to analyze the chromosome CNV of epithelial cells using endothelial cells as a reference (**Supplementary Figure S1**). According to CNV level, malignant cells was distinguished from EPCs. After that, we re-clustered 3,076 malignant cells, and annotated them according to each cell marker gene, and identified four malignant cell subtypes: C0 *TRPM4*+ malignant cells, C1 *SLPI*+ malignant cells, C2 *MIR205HG*+ malignant cells and C3 *NEFH*+ malignant cells (**Figure 3A**). We employed the UMAP plot combined with a cellular proportion pie chart to illustrate the relative distribution of different subgroups (THG and TLG) within the four malignant cell subtypes (**Figure 3B**). Additionally, we visualized these cells based on tissue types (**Figure 3C**). The results demonstrated that, compared to other subtypes, the C1 and C3 malignant cell subtypes exhibited a higher proportion of THG tissue. Additionally, a stacked bar graph revealed that THG tissue predominantly comprised the C1 and C3 subtypes, in contrast to TLG tissue (**Figures 3D, E**). Therefore, we hypothesized that the heterogeneity between these two tissue types may be associated with the C1 and C3 subtypes. Similarly, the Ro/e preference plot indicated a higher cell abundance of C1 and C3 subtypes in THG tissue, further substantiating our conclusion (**Figure 3F**). **Figure 3G** showcased the differential expression of the top five marker genes in the malignant cell subtypes, visualized using the volcano plots (**Figure 3H**).

Next, we showed the results of CNVscore, nFeature-RNA and nCount-RNA of different tissue types and malignant cell subtypes by violin plots (**Figures 3I, J**). It was found that the CNVscore of THG was higher than that of TLH, which was consistent with the biological process of PCa tissue. It is noteworthy that both the C1 and C3 subtypes exhibited higher CNV scores. Additionally, compared to the C1 subtype, the C3 subtype with high NEFH expression showed elevated levels of nCount-RNA and nFeature-RNA. Therefore, we inferred that the C3 subtype’s malignancy level might have been higher.

Subsequently, we determined that the subtypes shared specific biological functions through the enrichment analysis of a variety of malignant cells. For instance, transition metal homeostasis was associated with both C1 and C3 subtypes. Furthermore, the C3 subtype was enriched in the cholesterol metabolism process (**Figures 3K–M**). We also illustrated the metabolism pathways using bubble plot and discovered that the C3 subtype was substantially enriched in the metabolic pathways of glutathione metabolism and fatty acid biosynthesis (**Figure 3N**).

### Unveiling the development and differentiation characteristics of malignant cell subtypes through pseudotime analysis

**Figure 4:**
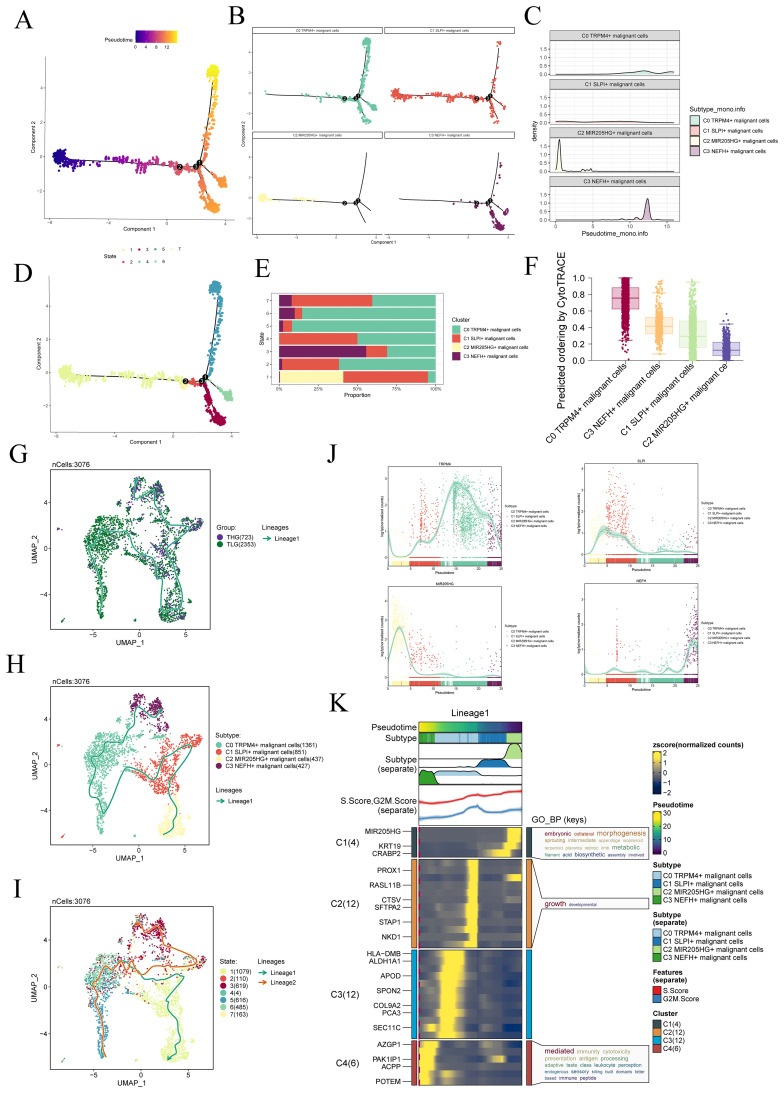
Slingshot analysis of malignant cell subtypes. **(A)** The Monocle analysis was utilized to infer the developmental trajectory of malignant cells. **(B)** Cells were colored based on pseudotime and visualized to show the position of different cell types along the developmental trajectory. **(C)** The ridge plot depicted the dynamic changes in the cell state of different cell subtypes. **(D, E)** The pseudotime was divided into seven-time states (States 1-7) based on the time order, and the slingshot analysis inferred the differentiation trajectories of different cell subtypes at each time stage. The stacked bar graph showed the proportions of cell subtypes in each of the seven-time stages. **(F)** The Cytotrace analysis was employed to rank the stemness of malignant cell subtypes. **(G–I)** The UMAP plots displayed the slingshot differentiation trajectories of different malignant cell subtypes, different tissue types, and different time stages. The solid lines represented the differentiation trajectories, and the arrows indicated the direction of differentiation (from naive to mature). **(J)** Differential expression patterns of marker genes for four malignant cell subtypes during the differentiation process. **(K)** The GO-BP enrichment analysis confirmed the biological processes corresponding to the pseudotime trajectory of malignant cell subtypes.

To understand the source and development of cancer cells, we extensively studied the intricate lineage and advancement of malignant cells utilizing the Monocle software. It was easy to see in **Figures 4A–E** that the C2 *MIR205HG+* malignant cells were mostly in the early stages of differentiation, more specifically in state 1 along the time axis. Conversely, the C3 *NEFH+* malignant cells were in the last stage of development, predominantly found within state 3 throughout the temporal trajectory. Afterwards, we utilized the CytoTRACE technique to evaluate the differentiation and developmental correlation between several subtypes of tumor cells. The findings indicated that C3 *NEFH+* malignant cells displayed elevated cellular stemness, as seen in **Figure 4F**. Combining the Slingshot analysis, we discovered a differentiation boundary transitioning from TLG to THG tissue types, indicating a higher malignant degree among the cells at the terminal stage of differentiation (**Figure 4G**). Malignant cells often possess self-renewal capability and differentiation potential. Thus, as the tumor advances, malignant cells in the last stage of differentiation tend to have greater cellular stemness, in line with the results obtained from the CytoTRACE investigation. Furthermore, the progression of cancerous cell subtypes can be described in the order of C2→C1→C0→C3. Additionally, the slingshot analysis of time states showed that State 3 was positioned at the end of one of the differentiation branches, with a greater proportion of C3 subtypes within state 3. This finding further supports the conclusions drawn from the Monocle analysis (**Figures 4H, I**).

Consistent with those findings, the genes *TRPM4*, which serve as markers for the C0 subtype, and *NEFH*, which serve as markers for the C3 subtype, were predominantly expressed during the mid-late stage of the developmental trajectory. On the other hand, the expression levels of *SLPI*, a marker for the C1 subtype, and *MIR205HG+*, a marker for the C2 subtype, were initially high but declined over time (**Figure 4J**). In addition, we conducted GO-BP enrichment analysis on the DEGs linked to the malignant cell subtypes. The analysis revealed that these genes were mainly enriched in biological processes related to immunity, cytotoxicity, antigen, processing, and other similar activities (**Figure 4K**).

### Cell-cell communication and visualization of the PTN signaling pathway

**Figure 5:**
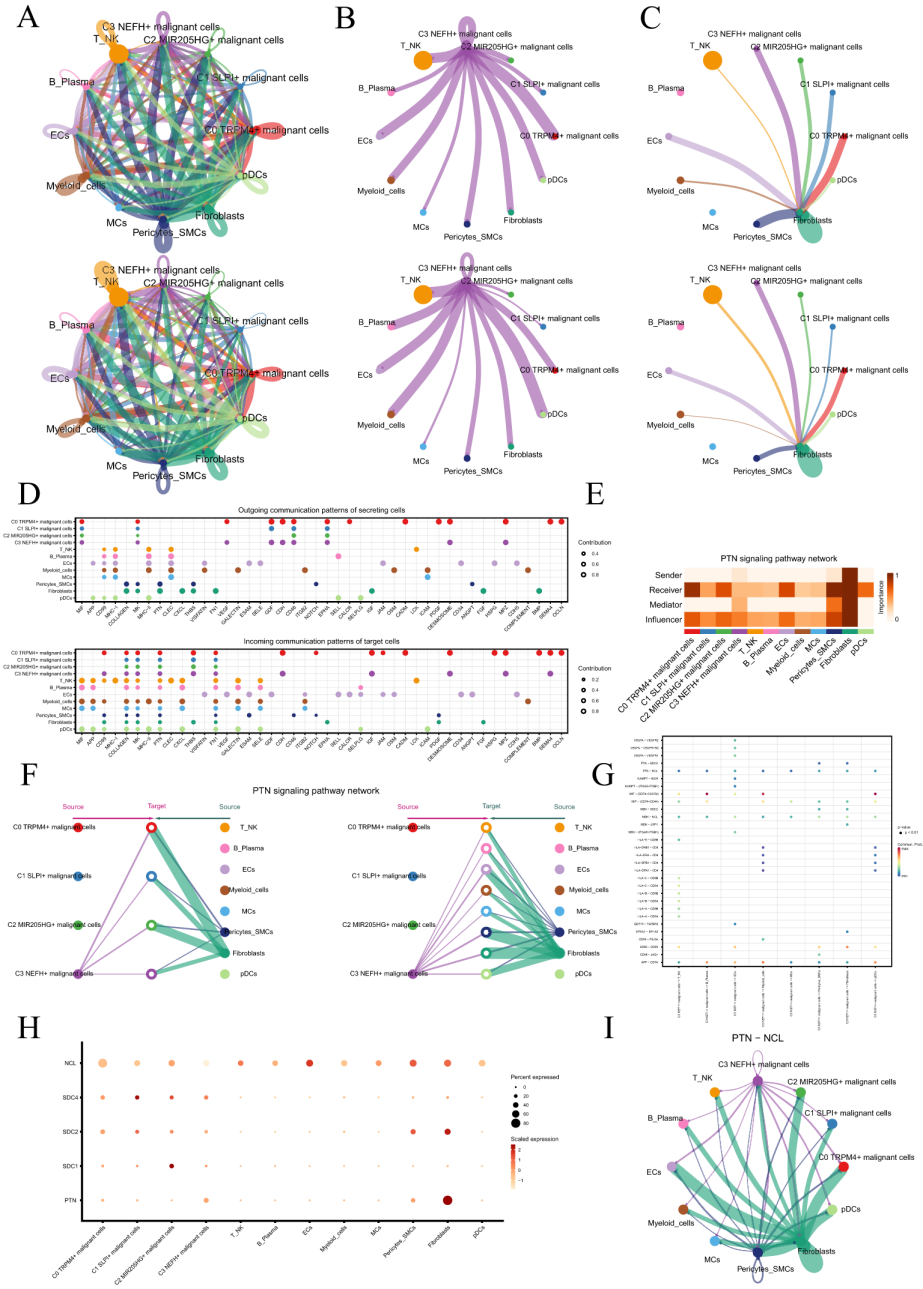
Cellular communication landscape in PCa. **(A)** Circle graphs displayed the number (upper) and intensity (lower) of interactions among all cells in PCa. The size of each circle was proportional to the number of cells in each group, and the edge width represented the communication probability. **(B)** Circle graphs displayed the number (upper) and intensity (lower) of interactions of C3 *NEFH+* malignant cells as the source with other cells. **(C)** Circle graphs displayed the number (upper) and intensity (lower) of interactions of fibroblasts as the target with other cells. **(D)** Bubble plots compared the outgoing communication patterns of secretory cells (upper) and the incoming communication patterns of target cells (lower). The size of each dot was proportional to the contribution score calculated by pattern recognition analysis. Higher contribution scores indicated richer signaling pathways in the corresponding cell group. **(E)** Heatmap displayed the centrality scores of the PTN signaling pathway. **(F)** Hierarchical graph depicted the interactions between C3 *NEFH+* malignant cells and other cell types in the PTN signaling pathway. **(G)** Comparative Analysis of Significant Ligand-Receptor Pairs in the Interaction of C3 *NEFH+* Malignant Cells with Other Non-malignant Cell Types. The color of the dots represents the probability of communication between specific ligand-receptor pairs across the sender cell clusters and the recipient cell clusters. The ligand is denoted as the former and the receptor as the latter, separated by a hyphen. **(H)** Bubble plot displayed the interactions between cells in the PTN signaling pathway. **(I)** Circle plot showed the communication network of PTN-NCL ligand-receptor pairs with tumor cells as the receiver.

We utilized CellChat to infer and analyze communication between tumor cell subtypes and other cell types from single-cell data (**Supplementary Table S3**). The number and intensity of interactions between all cell types in PCa samples were comprehensively summarized (**Figure 5A**). It was found that compared with other types of cells, C3 *NEFH+* malignant cells had a more significant effect on pericytes-SMCs and fibroblasts. The circle graphs quantified the number and intensity of interactions between all cells with C3 *NEFH+* malignant cells as the signal source and fibroblasts as the target respectively (**Figures 5B, C**). The results showed that there was a strong intercellular communication network between C3 *NEFH+* malignant cells and fibroblasts.

Next, we identified the ligand-receptor signals associated with the communication pathway (**Figure 5D**) to determine the primary afferent and efferent signals related to the C3 *NEFH+* malignant cell subtype and other cells. The findings indicated that the primary ligands associated with the output of C3 *NEFH+* malignant cells, when employed as signal senders, were MIF, MK, GDF, and CD46. As signal receivers, the fibroblast-related receptors mainly included PTN, CD99, and PDGF.

Subsequent analysis revealed potential connections to the PTN signaling pathway network. Through network centrality analysis of the inferred PTN signaling network, we found that C3 *NEFH+* malignant cells can act as signal senders within the PTN pathway. Fibroblasts, on the other hand, can function both as signal sender promoting their transformation into cancer-associated fibroblasts (CAFs) and as signal receiver, mediator, and influencer interacting with C3 NEFH+ malignant cells (**Figure 5E**). Notably, C3 *NEFH+* malignant cells demonstrated the ability to engage in paracrine interactions with fibroblasts, resulting in a substantial communication intensity between these cell populations (**Figure 5F**). In addition, we compared the receptor-ligand interaction between C3 NEFH+ malignant cells and other cell types and found that when this subtype interacted with fibroblasts, the ligand receptor had a high communication probability with PTN-NCL (**Figures 5G, H**). Additionally, a circle graph further confirmed that the interactions between C3 *NEFH+* malignant cells and fibroblasts could be mediated through the receptor-ligand pairs within the PTN signaling pathway, specifically involving PTN-NCL (**Figure 5I**).

Essentially, our study provided profound insights into the intricate interactions between fibroblasts and malignant cell subtypes in PCa. This relationship is likely closely linked to the transformation of fibroblasts into CAFs, which promotes the progression of PCa.

### Identification and analysis of TFs regulatory modules

TFs can directly interact with the genome and regulate gene transcription by binding to specific nucleotide sequences upstream of the target gene. This interaction plays a significant role in determining the biological functions of cells.

**Figure 6:**
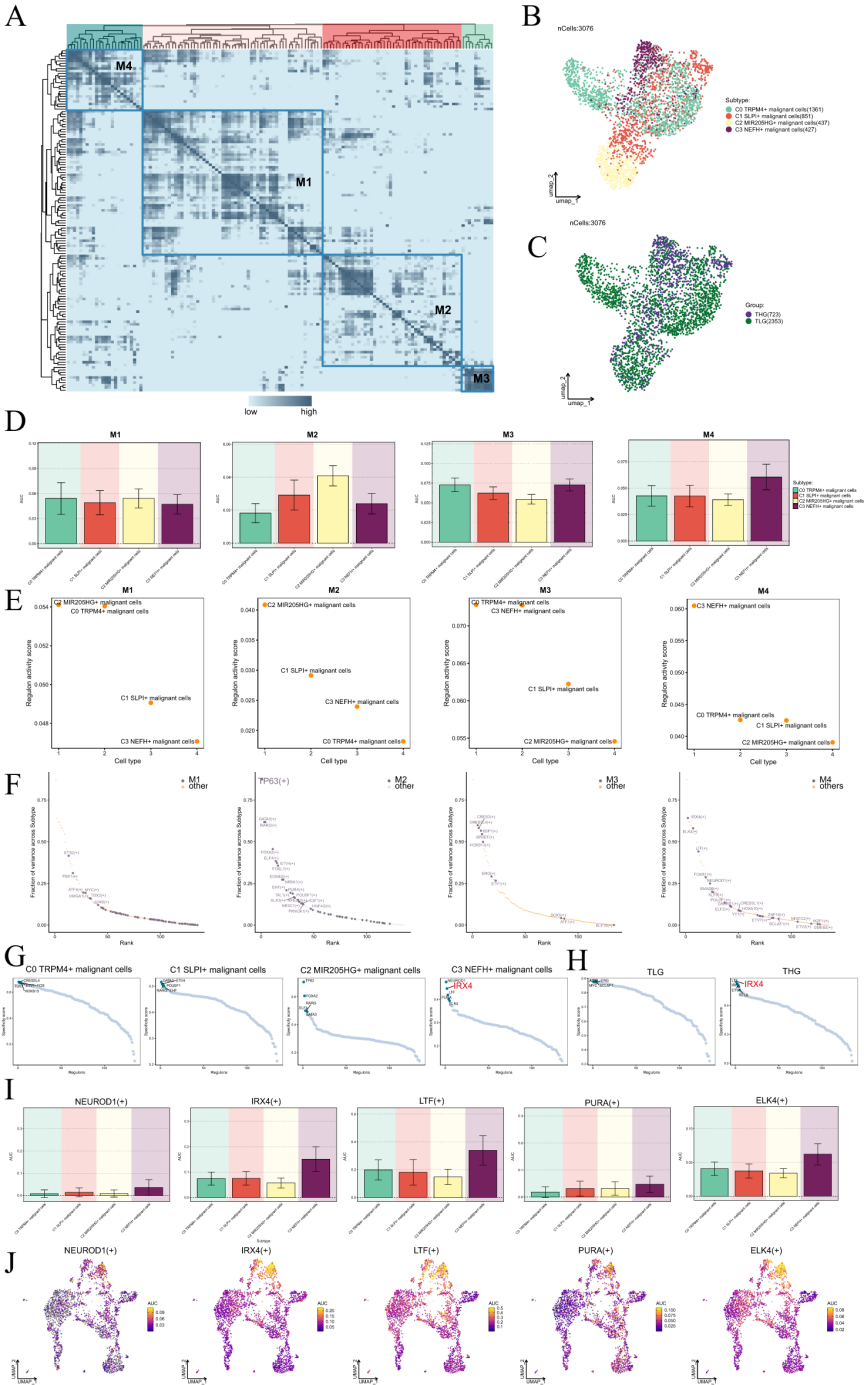
Identification of gene regulatory networks in C3 *NEFH+* malignant cells. **(A)** Heatmap displayed the identification of four regulatory modules in malignant cell subtypes based on SCENIC regulatory rule modules and AUCell similarity scores. **(B, C)** UMAP plots colored and visualized all malignant cells based on the activity scores of regulatory modules, respectively, according to cell subtypes and tissue types. **(D)** Bar graphs provided a visual comparison of the AUC values of TFs in each module across different malignant cell subtypes. **(E)** Scatter plots displayed the regulatory activity of TFs in each module across different malignant cell subtypes. **(F)** Scatter plot displayed the ranking of TFs based on the fraction of variance across subtype in each module. **(G, H)** Rank the regulatory factors of different malignant cell subtypes and tissue types based on the regulatory specificity score. **(I, J)** Bar graphs depicted the AUC value of the top five TFs in C3 *NEFH+* malignant cells across different malignant cell subtypes. UMAP plots visualized the distribution of these TFs.

To begin with, we employed the SCENIC and connection specificity index matrix to classify prostate malignant cells into four regulatory modules (M1, M2, M3, M4) based on the similarity of AUCell score rules (**Figure 6A**). Subsequently, we conducted dimensionality reduction and clustering analyses considering various subtypes and tissue types (**Figures 6B, C**).

Through a comparison of the expression levels and regulatory activities of TFs within each module and the malignant cell subtypes, we identified that the TFs in the M4 module predominantly regulated C3 *NEFH+* malignant cells (**Figures 6D, E**). Within the M4 module, we found that IRX4 exhibited the highest fraction of variance across the subtypes, indicating its prominent role in explaining a significant portion of the data variability within the M4 module (**Figure 6F**). Importantly, IRX4 demonstrated a high specificity score in C3 *NEFH+* malignant cells and THG (**Figures 6G, H**). This suggested a strong and specific regulatory relationship between IRX4 and its target genes, highlighting its potential as a biomarker or therapeutic target.

Finally, we visualized the expression levels of five key regulatory factors (NEUROD1, IRX4, LTF, PURA, and ELK4) in the C3 subtype (**Figures 6I, J**). We observed that the expression of IRX4 in the C3 subtype was significantly higher compared to other malignant cell subtypes. To further investigate the relevance of these TFs, we performed survival analysis using Kaplan-Meier and AUC curves (**Supplementary Figures S2A–F**). Interestingly, our results indicated that high expression of IRX4 might be associated with a poorer prognosis in PCa. Nevertheless, the specific mechanism by which IRX4 influences PCa remains unclear. Therefore, conducting *in vitro* functional experiments to validate the impact of IRX4 on PCa cells is imperative.

### *In vitro* experimental verification

**Figure 7:**
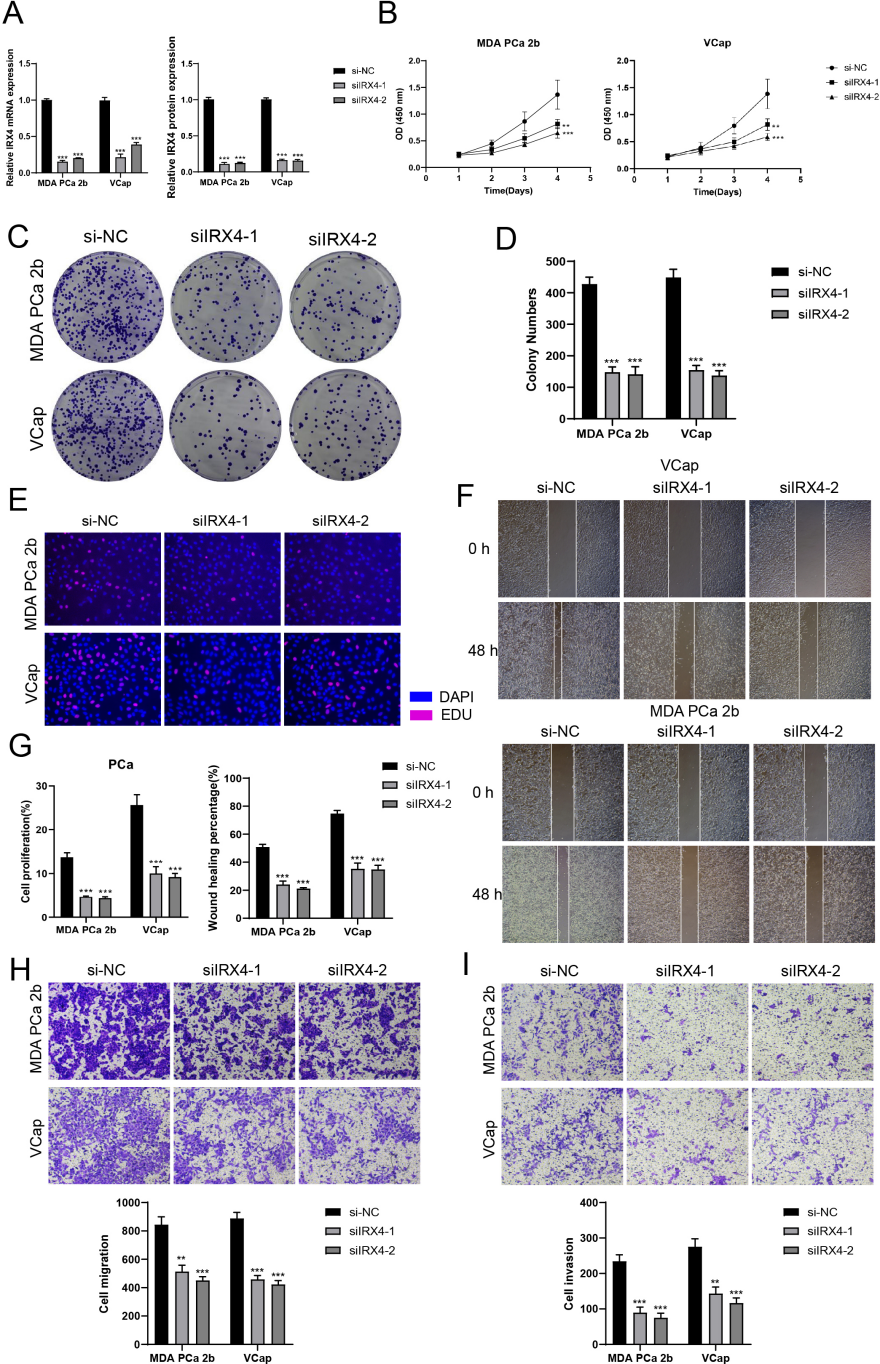
*In vitro* experiments confirmed the effects of IRX4 knockdown. **(A)** Decreased mRNA and protein expression levels after IRX4 knockdown. **(B)** CCK-8 assay showed a significant decrease in cell viability after IRX4 knockdown compared to the control group. **(C, D)** Colony formation assay demonstrated a significant decrease in colony numbers after IRX4 knockdown. **(E)** EDU staining experiment confirmed the inhibitory effect of IRX4 knockdown on cell proliferation. **(F)** Wound healing assay showed that IRX4 knockdown inhibited cell migration. **(G)** Bar graph displayed a significant decrease in cell proliferation and migration abilities after IRX4 knockdown. **(H, I)** Transwell assay showed that IRX4 knockdown suppressed the migration and invasion abilities of tumor cells in MDA PCA 2b and VCap cell lines. **P<0.01, ***P<0.001.

To further investigate the role of IRX4 in PCa, we conducted *in vitro* experiments using MDA PCA 2b and VCap cell lines. Initially, we knocked down IRX4 and assessed the mRNA and protein expression levels before and after knockdown. Our findings revealed a significant decrease in mRNA and protein expression levels in both cell lines compared to the control group (**Figure 7A**). Moreover, there was a noticeable decrease in cell viability following the knockdown (**Figure 7B**).

Subsequent colony experiments demonstrated a significant reduction in the number of cells after IRX4 knockdown (**Figures 7C, D**). Additional EDU experiment confirmed that the knockout of IRX4 partially inhibited cell proliferation (**Figure 7E**). Furthermore, the wound healing assay and Transwell assay indicated a substantial decrease in cell migration after IRX4 knockdown (**Figures 7F–H**), and the cell invasion capability also decreased (**Figure 7I**).

Collectively, these results indicate that the knockdown of IRX4 can inhibit the activity, migration, and proliferation of tumor cells, thereby impeding tumor growth.

### Construction and correlation analysis of risk prediction model

**Figure 8:**
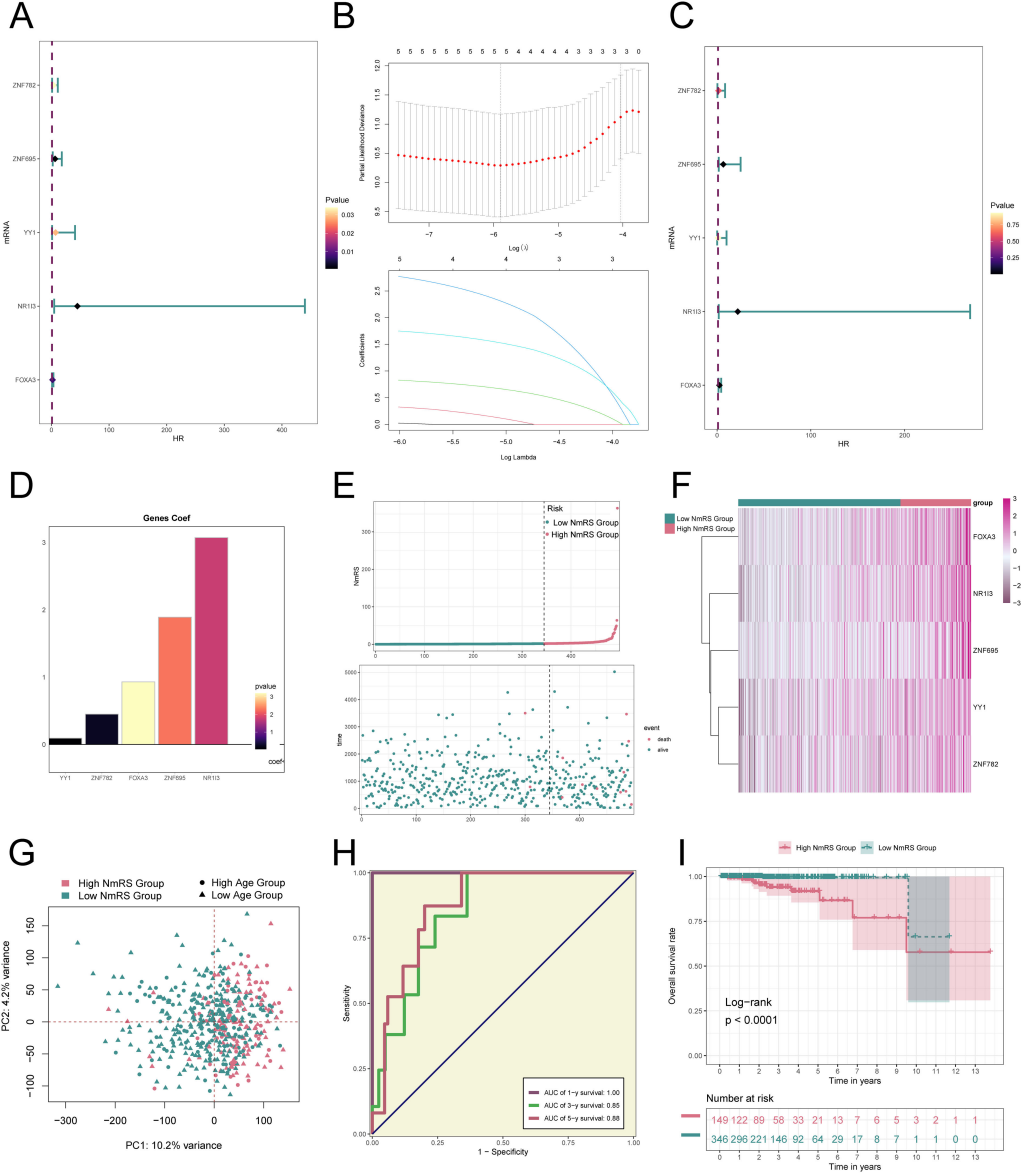
Construction and validation of the *NEFH+* malignant cells risk score (NmRS) model. **(A)** Forest plot of univariate Cox regression analysis showed genes with significant differences (HR<1: protective factors, HR>1: risk factors). **(B)** LASSO regression analysis identified five prognostic-related genes (non-zero regression coefficients). The optimal parameter was determined through cross-validation (upper plot), and the LASSO coefficient curve was determined using the optimal lambda (lower plot). **(C)** Forest plot displayed the results of multivariate Cox regression analysis. **(D)** Bar graph showed the Coef values of the genes used for model construction. **(E)** Curve plot displayed the risk scores of the high NmRS group and low NmRS group (upper plot), and scatter plot showed the survival/death events over time in the two groups (lower plot). **(F)** Heatmap displayed the differential expression of model genes, with color scale based on normalized data. **(G)** Scatter plot showed the distribution of genes along PC1 and PC2 in the high NmRS group and low NmRS group. **(H)** Sensitivity and specificity of 1-year, 3-year, and 5-year outcomes were evaluated using ROC curves and AUC values. **(I)** Kaplan-Meier curves displayed the survival differences between the high NmRS group and low NmRS group.

We developed a prognostic model to investigate the clinical significance of the NEFH+/IRX4 regulatory network. Initially, we performed univariate Cox regression analysis to identify genes significantly associated with prognosis (**Figure 8A**). To address the issue of multicollinearity among these genes, we employed LASSO regression analysis for further selection (**Figure 8B**). Subsequently, a multivariate Cox regression analysis was conducted, resulting in the identification of five genes related to prognosis. The coef values for these genes were calculated (**Figures 8C, D**). The findings revealed that *ZNF782, ZNF695, YY1, NR1I3*, and *FOXA3* were unfavorable prognostic factors.

To further investigate the differences between different scoring groups, we performed an analysis of DEGs. Based on the optimal cut-off value of *NEFH+* malignant cell score, patients in the TCGA cohort were categorized into two groups: the high NmRS group and the low NmRS group (NmRS: *NEFH+* malignant cells risk score). It was observed that higher scores were associated with worse prognosis. Curve and scatter plots were utilized to illustrate the differences in risk scores, survival, and outcomes between the two groups, clearly indicating that the high NmRS group was associated with a poorer prognosis (**Figure 8E**). Furthermore, a heatmap was generated to display the differential expression of the five genes between the high and low NmRS groups (**Figure 8F**). Principal component analysis demonstrated that PC1 (high NmRS group) accounted for 10.2% of the total variance in all principal components, while PC2 (low NmRS group) accounted for 4.2% of the total variance (**Figure 8G**). Additionally, the ROC curve provided an intuitive visualization of the AUC values predicted by the TCGA cohort at 1 year, 3 years, and 5 years, demonstrating the predictive value of the model (**Figure 8H**). The Kaplan-Meier survival curve further confirmed the conclusion that the high NmRS group had a worse survival outcome, with a *p-value* less than 0.0001 (**Figure 8I**).

**Figure 9:**
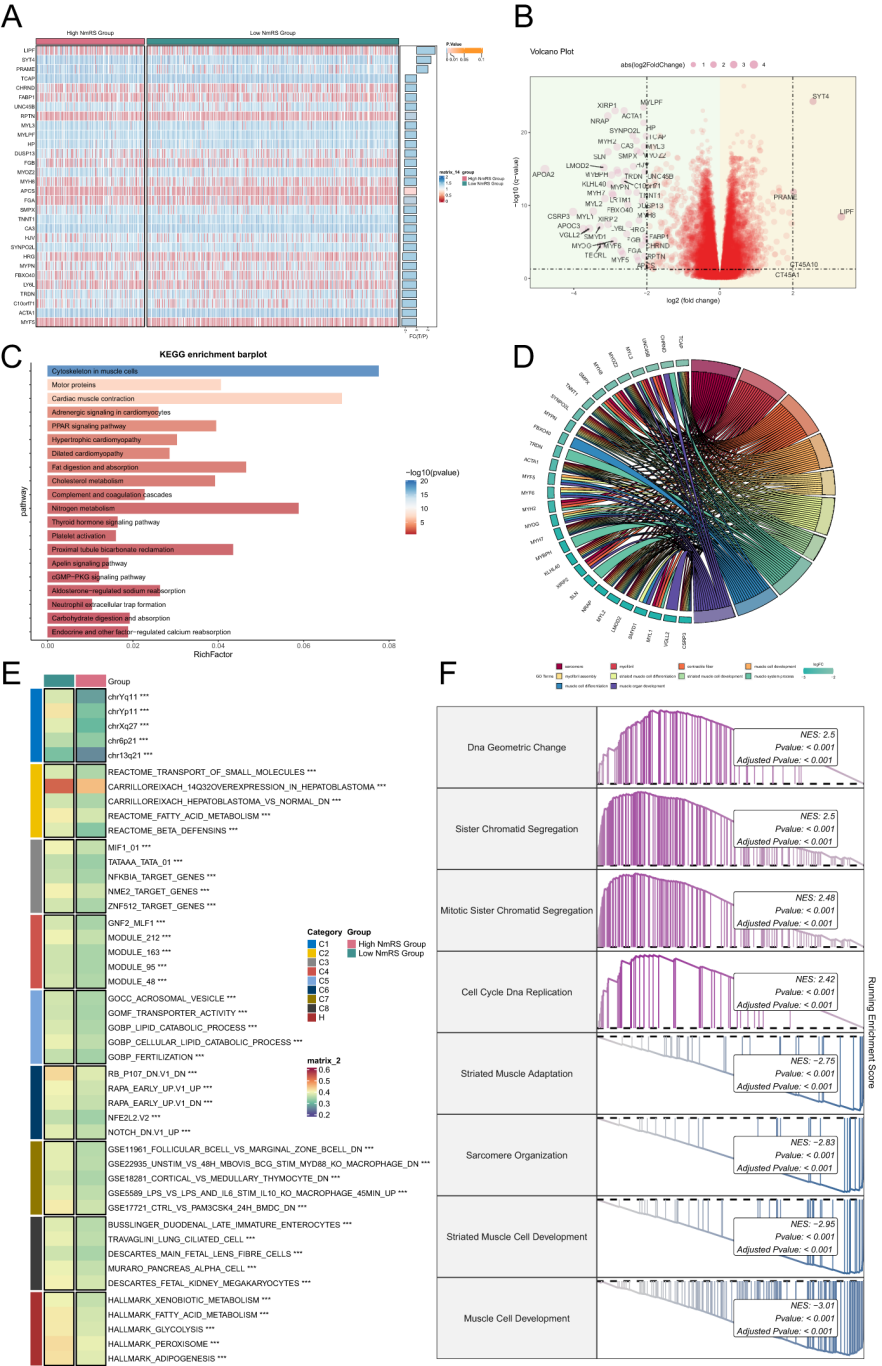
Enrichment analysis of DEGs and GSVA analysis results. **(A)** Heatmap displayed the expression of the top 30 DEGs in the high and low NmRS groups. **(B)** Volcano plot showed the upregulated and downregulated DEGs in the high and low NmRS groups. **(C)** Bar graph displayed the results of KEGG pathway enrichment analysis of DEGs in the high and low NmRS groups. **(D)** Chord plot displayed the results of GO enrichment analysis of DEGs in the high and low NmRS groups. **(E)** Heatmap illustrated the results of GSVA enrichment analysis of DEGs in the high and low NmRS groups. **(F)** Detailed description provided GSEA enrichment results for DEGs in different pathways. ***P < 0.001.

To elucidate differential gene expression and associated biological processes between high and low groups, we employed visualization and enrichment analysis techniques. Initially, a heatmap was utilized to depict gene expression of the top thirty genes (**Figure 9A**), while a volcano plot showcased differential gene up-regulation and down-regulation (**Figure 9B**).

Subsequently, various enrichment methods were employed to delve deeper into the related biological processes. KEGG analysis indicated predominant enrichment of differential genes in cytoskeleton in muscle cells, motor proteins, and cardiac muscle contraction (**Figure 9C**). Conversely, GO analysis revealed enrichment in myofibril and muscle cell development (**Figure 9D**). Furthermore, GSVA enrichment analysis was conducted on the gene set comprising the prediction model, as represented in the heatmap (**Figure 9E**). Lastly, GSEA analysis was performed on the initial 30 up-regulated and down-regulated genes. Up-regulated genes exhibited enrichment in DNA geometric change, sister chromatid segregation, mitotic sister chromatid segregation, cell cycle DNA replication, down-regulated genes were primarily enriched in striated muscle adaptation, sarcomere organization, striated muscle cell development, and muscle cell development (**Figure 9F**).

### Analysis of immune infiltration, mutation, and drug sensitivity

**Figure 10:**
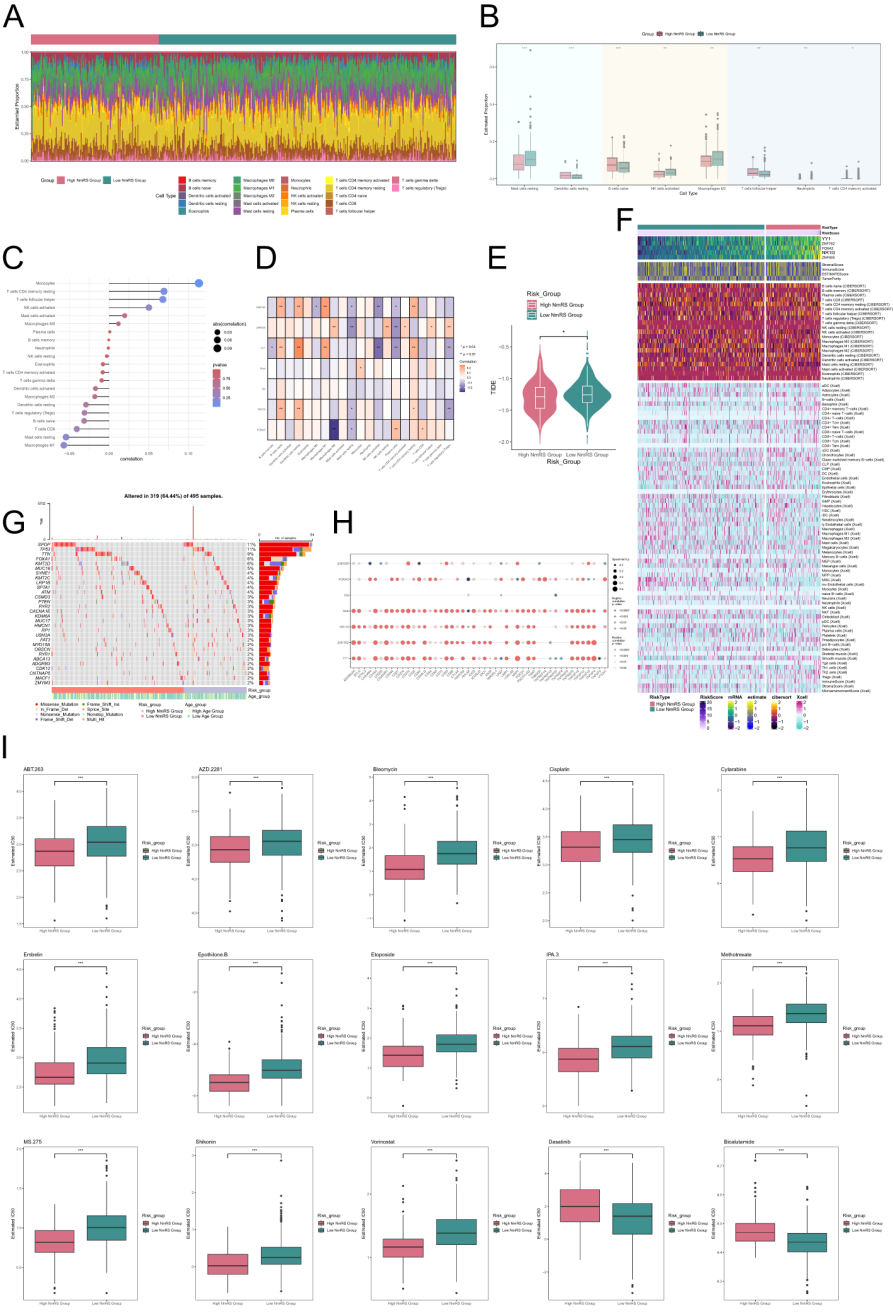
Analysis of immune infiltration differences and drug sensitivity in different risk groups of C3 *NEFH*+ malignant cells. **(A)** Stacked bar graph displayed the distribution of twenty-two immune cells among different risk score groups. **(B)** Box plot showed the differences in eight types of immune cells between high and low NmRS groups. **(C, D)** Lollipop chart and heatmap displayed the correlation analysis between immune cells and risk scores, model genes, and OS. **(E)** Differences in TIDE levels were shown between high and low NmRS groups. **(F)** Heatmap displayed the differences in infiltration levels of immune cells calculated using CIBERSORT and Xcell, as well as the model genes, StromalScore, ImmuneScore, ESTIMATScore, and Tumor Purity between the high and low NmRS groups. Color scale was based on standardized data. **(G)** Calculation of the correlation between DEGs and tumor mutation burden, and waterfall plot showed the results in the high and low-risk score groups. **(H)** Bubble plot showed the correlation between model genes, risk scores, OS, and immune checkpoint-related genes. **(I)** Drug sensitivity analysis. *P < 0.05, **P < 0.01, ***P < 0.001.

To investigate immune cell composition differences in NmRS with varying risk scores, we employed the CIBERSORT algorithm for analyzing twenty-two immune cell types in TCGA database PCa patients, as illustrated in **Figure 10A**. The proportions of eight immune cell categories were presented in **Figure 10B**, revealing distinctions between the two groups via a box plot. Notably, higher infiltration of naive B cells, T cells CD4 memory resting, and T cells follicular helper was observed in the high NmRS group, implying potential immune reactions associated with immune escape.

Subsequently, we assessed the correlation between immune cells and NmRS, as depicted in **Figure 10C**. The findings indicated a significant positive correlation between NmRS and macrophages, T cells CD4 memory resting, and T cells follicular helper, while a significant negative correlation was observed between NmRS and M1 macrophages. A heatmap visualization was employed to depict the correlation analysis among immune cells, modeling genes, OS, and risk score (**Figure 10D**). Moreover, differences in TIDE values between the two groups were evident (**Figure 10E**).

Additionally, **Figure 10F** displayed a heatmap showcasing variations in modeling genes, StromalScore, ImmuneScore, ESTIMATEScore, TumorPurity, and immune cell infiltration levels between the high NmRS and low NmRS groups, computed using the CIBERSORT and Xcell algorithms.

Next, we presented the correlation between DEGs and tumor mutation load using a waterfall diagram for the high and low-risk groups (**Figure 10G**). Furthermore, the correlation between immune checkpoint-related genes, modeling genes, risk score, and OS was displayed in a bubble plot (**Figure 10H**). The results revealed a strong positive correlation between *NR1I3, ZNF782, YY1*, and most immune checkpoints.

Finally, through drug sensitivity analysis, we identified potential clinical efficacy of certain drugs for prognosis-related genes, including ABT.263, AZD.2281, Bleomycin, Cisplatin, Cytarabine, Embelin, Epothilone.B, Etoposide, IPA.3, Methotrexate, MS.275, Shikonin, and Vorinostat. However, for the low-risk group, Dasatinib and Bicalutamide were found to be effective (**Figure 10I**).

## Discussion

PCa is a prevalent malignancy of the male reproductive system, and its treatment continues to face numerous challenges, particularly in the management of metastatic disease. Treatment resistance is one of the primary factors leading to therapeutic failure in PCa, significantly impacting patient prognosis (78). Research has shown that high-grade PCa with Gleason scores of 8-10 exhibit rapid growth, increased metastatic risk, and heightened resistance to treatment (79). High-grade PCa exhibited more aggressive biological behavior, often characterized by faster growth and an earlier tendency to metastasize. The rapid progression of these tumors could have been linked to the genetic instability of tumor cells, the activation of the EMT process, and the inflammatory responses within the tumor microenvironment (80). The metabolic reprogramming in high-grade PCa likely involved changes in lipid metabolism. Studies indicated that prostate cancer cells were more dependent on fatty acid oxidation as the primary energy supply pathway, with relatively lower glucose uptake rates (81). To address these challenges, the rapid development of scRNA-seq in recent years has provided powerful tools for cancer immunology research. These technologies facilitate an in-depth analysis of cellular interactions within the TME and their roles in disease progression, thus offering new perspectives for exploring treatment strategies (82).

To elucidate the complexities of the PCa TME, we employed scRNA-seq to depict the overall landscape of the TME. Dimensionality reduction and clustering analyses revealed a significant increase in the proportion of tumor cells among various stromal cell types, including EPCs, pericyte-SMCs, fibroblasts, ECs, and myeloid-cells. Notably, PCa primarily originates from prostatic glandular epithelial cells. Furthermore, previous studies have indicated that stromal cells, such as fibroblasts and smooth muscle cells, are crucial components of the PCa microenvironment and are closely associated with the malignant transformation of EPCs and cancer progression (83).

Additionally, inflammatory responses are recognized as a significant hallmark of cancer. In PCa patients, the expansion of myeloid cells in peripheral blood is often correlated with a shortened tumor survival period and resistance to treatment (84). Studies have indicated that myeloid inflammatory cells play critical roles in promoting the progression of PCa and treatment resistance (85). This finding aligns with our conclusions and further underscores the interactions among various cellular components in the TME and their impact on PCa progression.

To reveal the intra-tumor heterogeneity of malignant epithelial cells, we categorized the obtained cells into four subtypes: C0 TRPM4+ malignant cells, C1 SLPI+ malignant cells, C2 MIR205HG+ malignant cells, and C3 NEFH+ malignant cells. TRPM4 was a calcium-activated, non-selective cation channel that was widely expressed in various organs, immune cells, and the central nervous system. It was involved in physiological processes such as circulation, immune response, cancer, and hormone secretion (86). Secretory leukocyte protease inhibitor (SLPI), a protein with broad anti-inflammatory and immunoregulatory functions, its expression had altered in diabetic nephropathy (DN), likely linked to its role in reducing inflammation and protecting the kidneys from damage (87). MIR205HG, a long non-coding RNA (lncRNA), played a key role in esophageal cancer, potentially influencing tumor progression by regulating processes such as cell proliferation, migration, invasion, and apoptosis, and it might serve as a potential diagnostic and therapeutic target for esophageal cancer (88). NEFH (Neurofilament Heavy Chain) is a neurofilament protein primarily found in axons of neurons, where it promoted efficient neural signal transmission and was linked to the diameter of nerve fibers, affecting the speed of signal conduction (89). Analysis of these subtypes revealed that the C3 NEFH+ malignant cell subtype is closely related to high-grade tumors and exhibits a higher CNV score, indicating greater malignancy.

Understanding the metabolic changes in cancer unveiled critical energy pathways, including glycolysis, oxidative phosphorylation, glutaminolysis, and lipid metabolism, which played essential roles in cancer cells and served as key targets for cancer therapies (90). Targeting metabolism was also recognized as an important approach to enhance the effectiveness of standard treatments such as chemotherapy, radiotherapy, and immunotherapy. For example, targeting the metabolism of tumor cells could counteract the metabolic adaptations induced by standard therapies, thereby improving their sensitivity (91). Metabolomics technology was valuable for identifying metabolic reprogramming pathways and key enzymes associated with disease progression and drug resistance, providing insights into their functions and molecular regulatory mechanisms. These findings could identify metabolic vulnerabilities related to disease and resistance, validating their potential as novel molecular targets for new drug development or combination therapies (92). In addition, our metabolic analysis showed significant enrichment of the C3 subtype in metabolic pathways, such as glutathione metabolism and fatty acid biosynthesis. Tumor cells typically exist in a reductive microenvironment, characterized by elevated levels of glutathione. Previous research has indicated that the baseline glutathione concentration in PCa cells is significantly higher than that in normal cells (93, 94). Moreover, excessive dietary fat intake is considered a major risk factor for PCa, with fatty acid metabolism playing an essential role in promoting the proliferation of PCa cells (95). Therefore, the enrichment of the C3 subtype in these metabolic pathways not only highlights its importance in the metabolic adaptation of PCa cells but may also reflect its potential role in tumor progression and resistance. These findings offer new avenues for metabolic intervention strategies targeting PCa.

Subsequently, through pseudotime analysis, we found that this subtype was located at the terminal end of the developmental trajectory, exhibiting higher stemness characteristics. We observed a differentiation line indicating a transition of tissue types from TLG to THG, suggesting that cells at the differentiation endpoint possessed higher malignancy. Notably, the distribution of the differentiation endpoint closely aligned with that of the C3 subtype. This observation led us to speculate on the intricate relationship between the C3 *NEFH+* malignant cell subtype and the progression of high-grade PCa. Moreover, the trajectories we identified provided significant insights into the biological implications of tumor progression. The alignment of the differentiation endpoint with increased malignancy highlighted how the cells’ developmental status could influence their aggressive behavior and overall tumor dynamics. By understanding these trajectories, we could better correlate specific stemness features with the transition to more aggressive cancer phenotypes. This emphasized the critical need to study the C3 subtype as a unique research entity, as it may serve as a key player in the advancement of high-grade PCa and its associated malignancy features. In the TME, the role of the tumor stroma is often perceived as dualistic, with the potential to both inhibit tumor development and promote its progression. In PCa, the mechanisms governing the interactions between epithelial and stromal cells remain insufficiently characterized compared to other malignancies. Fibroblasts, which are crucial components of the stroma, are prevalent in the TME and significantly influence tumor cell proliferation and migration through the secretion of various cytokines, matrix proteins, and growth factors (96). The TME of PCa was composed of a complex cellular ecosystem that included tumor cells, immune cells, and stromal cells, among other cell types. This heterogeneity was particularly evident in prostate cancer and was closely linked to patient prognosis. While heterogeneity in the TME was also observed in other cancers, such as breast and lung cancer, the cellular makeup and interaction patterns might have differed (97). In prostate cancer, the infiltration of regulatory T cells (Tregs) was associated with an immunosuppressive microenvironment in advanced disease, possibly induced by a FAP+ fibroblast subpopulation (98). Moreover, various cell subpopulations and transcriptional levels related to disease progression in prostate cancer were altered, which could have differed from the stromal changes noted in other cancer types (99). Notably, fibroblasts can differentiate into CAFs, which have been shown to play critical roles in tumor initiation and progression (99). The complexity of interactions between fibroblasts and PCa cells involves multiple signaling pathways, complicating our understanding of their interconnected relationships within the TME. To elucidate the potential interaction mechanisms between C3 *NEFH+* malignant cells and fibroblasts, we employed a systematic analysis of intercellular communications using the CellChat tool. Our results revealed a robust communication network between C3 *NEFH+* malignant cells and fibroblasts. Importantly, C3 *NEFH+* malignant cells were found to secrete the factor PTN, which interacts with fibroblasts via its receptor, NCL. This interaction is pivotal in facilitating the transformation of fibroblasts into CAFs, a process closely associated with tumor progression. These findings underscore the significant role of C3 *NEFH+* malignant cells in modulating the PCa microenvironment. The biological implications of our findings are profound. C3 *NEFH+* malignant cells appear to actively reconfigure the stromal landscape, thereby creating an environment conducive to tumor growth and metastasis. This insight suggests that targeting C3 *NEFH+* malignant cells may offer a promising therapeutic strategy. Disruption of the signaling pathways that mediate the interaction between these malignant cells and fibroblasts could lead to the development of novel treatments aimed at counteracting the supportive role of the stroma in tumor progression. From a therapeutic standpoint, targeting the PTN-NCL signaling axis may provide an innovative approach to inhibit CAF formation and thereby attenuate tumor aggressiveness. Such targeted therapies could be synergistically integrated into existing treatment protocols, potentially enhancing patient outcomes by addressing the TME’s influence on cancer progression. A key difficulty in drug development was ensuring the specificity and selectivity of the drug in order to reduce off-target effects. For instance, the knockdown of PTN in young mice resulted in defects in adult neurogenesis and cognitive dysfunction. This highlighted the need for careful consideration of the potential neurotoxic effects when developing drugs targeting the PTN-NCL signaling axis (100). Drug resistance was another significant challenge. In triple-negative breast cancer (TNBC) tissues that had relapsed after chemotherapy, the expression of PTN and its receptor PTPRZ1 was found to be elevated, which was closely linked to a poor prognosis (101). This suggested that tumors might acquire resistance by upregulating the PTN-NCL signaling axis during treatment, a factor that needed to be considered in drug development. Overall, these findings highlight the critical need for further research into the dynamic interactions within the TME and their implications for developing effective therapeutic interventions.

In our in-depth analysis of TFs, we identified key TFs in the C3 *NEFH+* malignant cell subtype, including NEUROD1, IRX4, LTF, POURA, and ELK4. Notably, IRX4 exhibited the highest subtype variance proportion in the M4 module, indicating its central regulatory role in C3 *NEFH+* malignant cells. Moreover, IRX4 demonstrated high specificity scores in both the C3 *NEFH+* malignant cell subtype and THG tissue, suggesting a strong regulatory relationship between this TF and its target genes. Consequently, IRX4 may serve as a potential biomarker or therapeutic target. Survival analysis results indicated that the expression level of IRX4 is likely associated with poorer prognosis (P<0.05). Previous research has confirmed that the knockdown of IRX4 can suppress stem-like characteristics and resistance to gefitinib in non-small cell lung cancer cells (102), although the specific mechanisms of IRX4 in PCa remain unclear. Thus, IRX4 may become a promising focal point in PCa research.

Early and accurate identification of high-grade PCa is crucial for advancing both basic research and clinical practice. However, existing methods are limited due to the lack of sensitive and specific biomarkers. Previous studies have evaluated at least ten prognostic models based on various gene signatures and machine learning algorithms; however, these models typically perform poorly in survival prediction (AUC < 0.6) (103). To address this gap, we constructed a new predictive model based on the top 100 marker genes of the C3 subtype, achieving good predictive accuracy. The final five genes associated with poor prognosis were *ZNF782, ZNF695, YY1, NR1I3*, and *FOXA3*. These findings provide new directions for cancer prediction and diagnosis.

Immune checkpoints play a critical role in regulating immune responses. Tumor cells often evade immune surveillance by upregulating immune checkpoints to inhibit local immune responses (104). Considering the widespread presence of immune cells in the TME of PCa, we analyzed differences in immune infiltration across different risk assessment categories. Compared to the low NmRS group, the high NmRS group exhibited significantly increased infiltration of naive B cells and resting CD4 memory T cells, likely reflecting an immune response associated with immune evasion (105). Interestingly, we found a negative correlation between the predictive model score and M1 and M2 macrophages, suggesting that tumors may promote the polarization of macrophages toward the M2 phenotype, thereby inhibiting M1 activity and leading to a dual reduction in the number of M1 and M2 macrophages. This change may be related to tumor progression and poor prognosis. Despite enhanced immune suppression and poorer prognosis in high-risk patient groups, they may exhibit greater sensitivity to chemotherapeutic agents such as Dasatinib and Bicalutamide, providing new research directions for subsequent clinical interventions. Tumor vaccines were found to have the potential to supplement conventional cancer therapies and targeted treatments. Their mechanism involved reprogramming the immune system to target and destroy cancer cells. These vaccines could have been one of the most promising strategies to overcome the inherent resistance in current cancer therapies (106). By examining the interactions between immune cells in the tumor microenvironment, new therapeutic approaches were developed, such as enhancing anti-tumor immune responses through modulation of the Th1/Th2 balance or influencing the function of Treg cells. The spatial architecture of the tumor microenvironment, particularly the tumor-stroma boundary, was emphasized for its impact on immune checkpoint blockade (ICB) efficacy. Targeting specific cell populations in defined spatial regions, like CXCL14+ CAFs, might have sensitized ICB responses (107). Although more research and clinical trials were needed to optimize immune microenvironment modulation for better therapeutic outcomes, the insights provided new avenues for developing treatments for PCa. Real-world studies (RWS) served as an important tool for assessing the efficacy and safety of drugs, especially in infectious diseases with multiple infection sites and complex pathogens. In the case of prostate cancer, this meant the need to evaluate the effectiveness of treatment regimens in a broader patient population (108). RWS showed that infections caused by multidrug-resistant Pseudomonas aeruginosa were difficult to treat, as many antibiotics were ineffective against this critical pathogen. This suggested that in prostate cancer, multidrug resistance (MDR) might also be a significant issue, highlighting the need for new treatment strategies to overcome it (109).

Single-cell sequencing technology has transformed biological research by enabling the detailed analysis of individual cells. This advancement has provided critical insights into cellular heterogeneity and the complex molecular mechanisms underlying diseases like PCa. In the realm of personalized treatment, single-cell analysis opens new avenues for understanding the tumor microenvironment and identifying specific cell types or subpopulations that contribute to disease progression or therapeutic resistance. By allowing a more nuanced view of tumor heterogeneity, single-cell analysis enhances our understanding of PCa biology. The identification of the C3 NEFH+ malignant cell subtype, in particular, presents a valuable opportunity for future clinical research. Investigating this subtype could lead to the development of targeted approaches for early screening and the optimization of treatment strategies. This includes the identification of potential biomarkers to enhance detection capabilities and the recognition of specific therapeutic targets to improve treatment effectiveness. Furthermore, comprehending the unique characteristics of the C3 NEFH+ subtype will facilitate patient stratification and enable personalized treatment decisions, ultimately leading to better patient outcomes. However, this study has several important limitations. Firstly, the sample size is relatively small, focusing primarily on single-cell data from a subtype of PCa patients, which may restrict the generalizability and applicability of the results. Secondly, the analytical methods used in this study mainly relied on single-cell sequencing and transcriptomic analysis, without considering other factors that may influence the outcomes. Therefore, future research should conduct multicenter studies with larger sample sizes to validate the potential roles of IRX4 and the constructed prognostic model in PCa. Additionally, incorporating proteomics and metabolomics approaches will provide deeper insights into the functional characteristics of specific subgroups and key molecules, thereby offering a more comprehensive basis for the early diagnosis and individualized treatment strategies for PCa. Through multi-omics analysis, we can better understand the biological mechanisms of tumors and identify potential therapeutic targets. In summary, our research focused on the diversity of epithelial cells in high-grade PCa at the individual cell level, further revealing the significance of IRX4 in this cancer type. Moreover, we identified several prognostically relevant genes, discovering a significant correlation between a higher NmRS and poorer prognosis. These findings not only enhance our understanding of the developmental mechanisms of PCa but also provide new opportunities for predicting and diagnosing this disease, with important clinical implications. Future studies should continue to explore these discoveries to advance research and treatment progress in PCa.

## Conclusion

This study utilized technology to delve into the complexities of the PCa TME, revealing the interactions between various cellular components and their influence on tumor progression. We identified the C3 *NEFH+* malignant cell subtype, which was associated with high-grade PCa and exhibits increased malignancy. The communication between malignant epithelial cells and fibroblasts through the PTN signaling pathway may be linked to the transformation of CAFs. TFs analysis identified key regulators, such as IRX4, which plays a central role in C3 *NEFH+* cells, with its expression level significantly correlating with patient prognosis. These findings provide new biomarkers and therapeutic targets for the early diagnosis and treatment of PCa. Furthermore, our survival prediction model based on C3 subtype marker genes demonstrated promising results, offering a new tool for clinical practice.

In summary, this research enhances our understanding of the PCa microenvironment and lays the groundwork for future therapeutic strategies and biomarker development. We anticipate that subsequent studies will validate these findings and explore their potential clinical applications, particularly focusing on cellular heterogeneity within the tumor microenvironment, elucidation of resistance mechanisms, and the development of early diagnostic biomarkers, thereby advancing our understanding of tumor biology.

## Data Availability

The single-cell data utilized for prostate cancer research is publicly available via PMID of 36750562 corresponding to GSE181294.

## Supplementary material

Supplementary Figure 1 The analysis of inferCNV. Using scRNA-seq data of endothelial cells to predict CNV. Red indicated amplification, while blue indicated deletion.

Supplementary Figure 2 The analysis of the C3 subtype marker gene and the top five TFs in bulk. Kaplan-Meier survival curves and ROC curves depicted the marked genes of C3 malignant cell subtype and the top five TFs (NEUROD1, IRX4, LTF, PURA, ELK4). **P* < 0.05, ***P* < 0.01, ****P* < 0.001, and *****P* < 0.0001. “ns” was used to say that there was no significant difference.
