# Correction: Unveiling the NEFH+ malignant cell subtype: insights from single-cell RNA sequencing in prostate cancer progression and tumor microenvironment interactions

**DOI:** 10.3389/fimmu.2025.1718125

**Published:** 2025-11-07

**Authors:** Jie Wang, Fu Zhao, Qiang Zhang, Zhou Sun, Zhikai Xiahou, Changzhong Wang, Yan Liu, Zongze Yu

**Affiliations:** 1 Department of Urology, The Second People’s Hospital of Meishan City, Meishan, Sichuan, China; 2 Department of Urology, China-Japan Union Hospital of Jilin University, Changchun, Jilin, China; 3 The First Clinical Medical College of Shandong University of Traditional Chinese Medicine, Jinan, China; 4 China Institute of Sport and Health Science, Beijing Sport University, Beijing, China; 5 Department of Urology, The First People’s Hospital of Jiangxia District, Wuhan, Hubei, China; 6 Department of Urology, Xiangyang Central Hospital, Affiliated Hospital of Hubei University of Arts and Science, Xiangyang, Hubei, China

**Keywords:** multi-omics, single-cell RNA sequencing, prostate cancer, tumor heterogeneity, precision medicine, drug discovery

There was a mistake in **Figure 1** and **Figure 7I** as published. This error occurred because placeholder images were used during the typesetting process to maintain layout consistency. Due to an oversight, the first placeholder image in the upper left corner of **Figure 7I** was not replaced with the correct image in time. In addition, since the **Figure 1** includes a schematic diagram of this figure, it also needs to be updated accordingly. The corrected **Figure 1** and **Figure 7** appears below.

**Figure 1:**
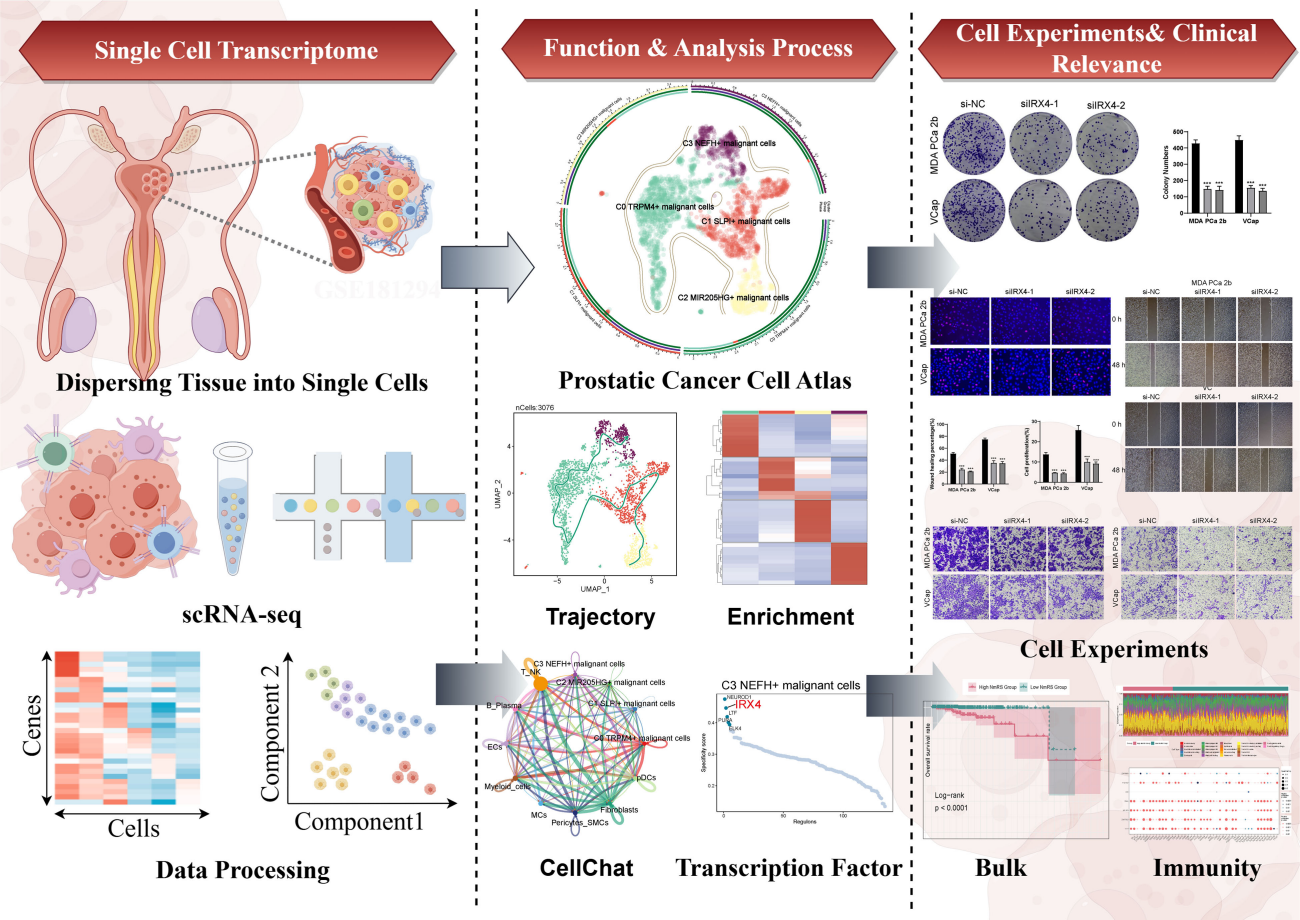
Graphical abstract. The analysis workflow for this research. We performed single-cell sequencing analysis on the GSE181294 dataset and identified a distinct C3 *NEFH*+ malignant cells subtype. Through pseudotime analysis, enrichment analysis, cell communication, and transcription factor regulation analysis, we revealed the significance of this subtype and confirmed the important role of the key TF (IRX4) through *in vitro* experiments. Prognostic and immune analyses provided guidance for clinical intervention and treatment.

**Figure 7:**
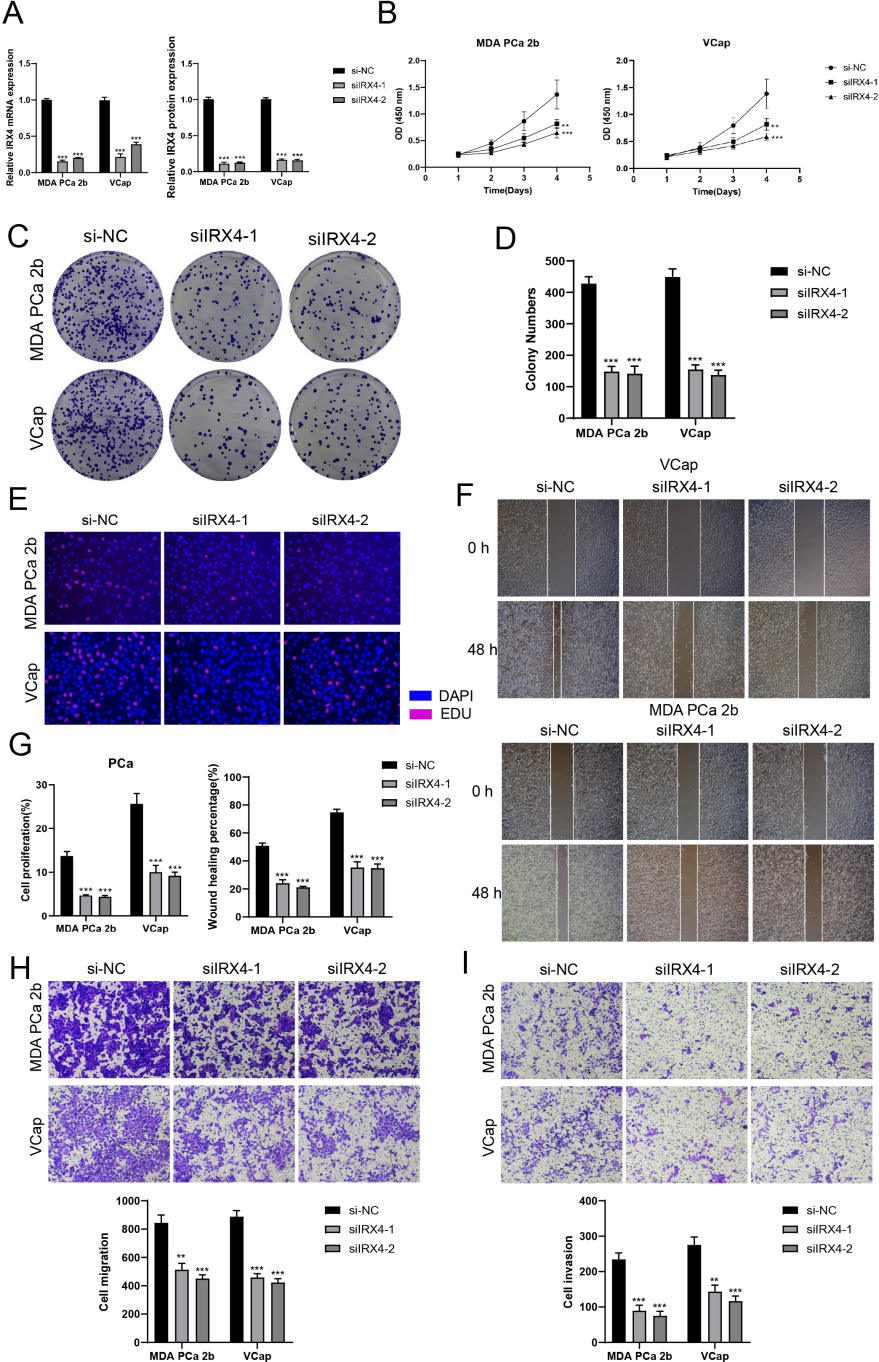
*In vitro* experiments confirmed the effects of IRX4 knockdown. **(A)** Decreased mRNA and protein expression levels after IRX4 knockdown. **(B)** CCK-8 assay showed a significant decrease in cell viability after IRX4 knockdown compared to the control group. **(C, D)** Colony formation assay demonstrated a significant decrease in colony numbers after IRX4 knockdown. **(E)** EDU staining experiment confirmed the inhibitory effect of IRX4 knockdown on cell proliferation. **(F)** Wound healing assay showed that IRX4 knockdown inhibited cell migration. **(G)** Bar graph displayed a significant decrease in cell proliferation and migration abilities after IRX4 knockdown. **(H, I)** Transwell assay showed that IRX4 knockdown suppressed the migration and invasion abilities of tumor cells in MDA PCA 2b and VCap cell lines. **P<0.01, ***P<0.001.

The original version of this article has been updated.

