# Supplementary figures and images for: Unveiling the NEFH+ malignant cell subtype: Insights from single-cell RNA sequencing in prostate cancer progression and tumor microenvironment interactions
Source: Front Immunol. 2024 Dec 20;15:1517679. doi: 10.3389/fimmu.2024.1517679 (PMC11695424; doi:10.3389/fimmu.2024.1517679)

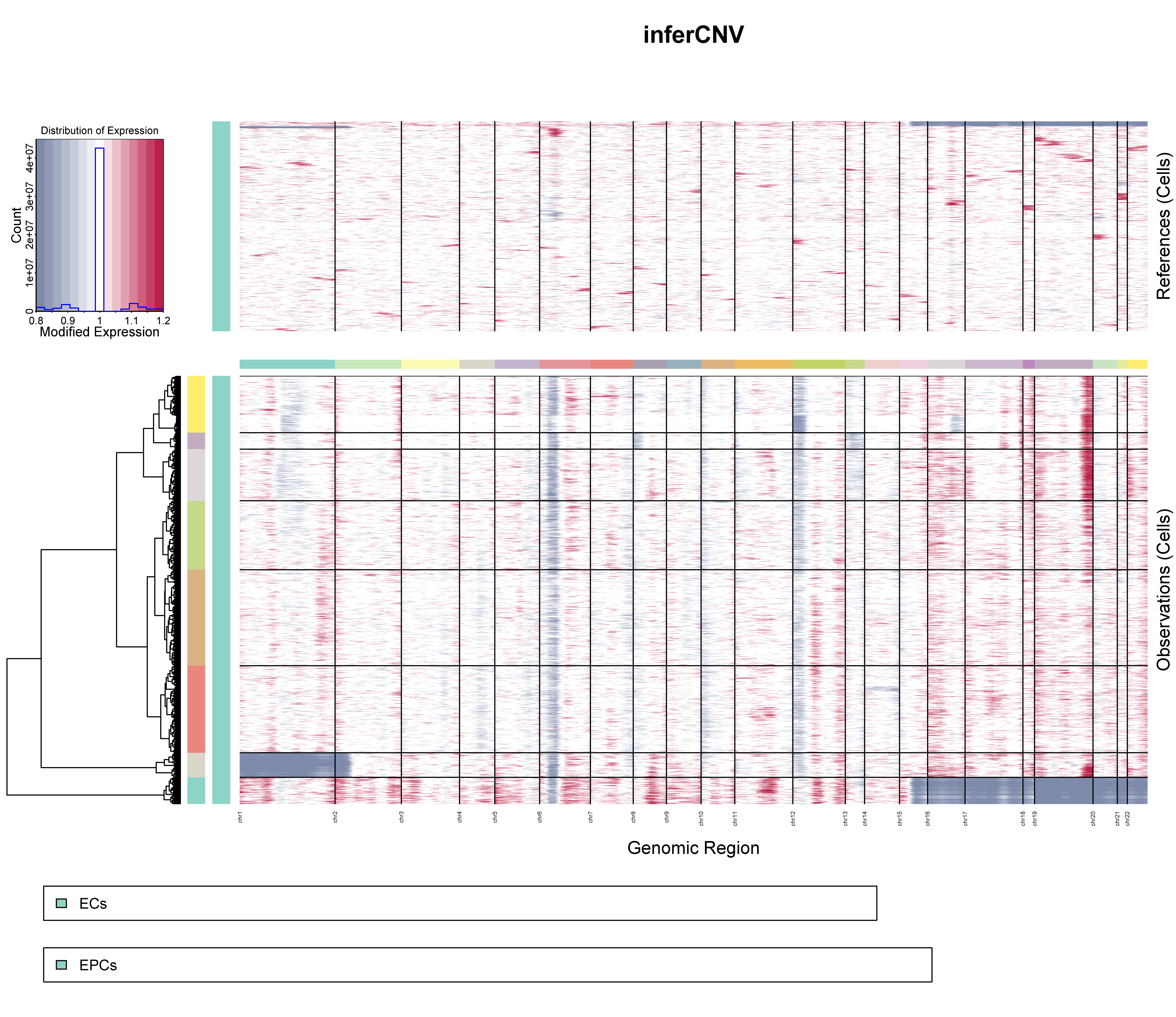

Supplement: Supplementary Figure 1 — The analysis of inferCNV. Using scRNA-seq data of endothelial cells to predict CNV. Red indicated amplification, while blue indicated deletion. [file Image1.tif]

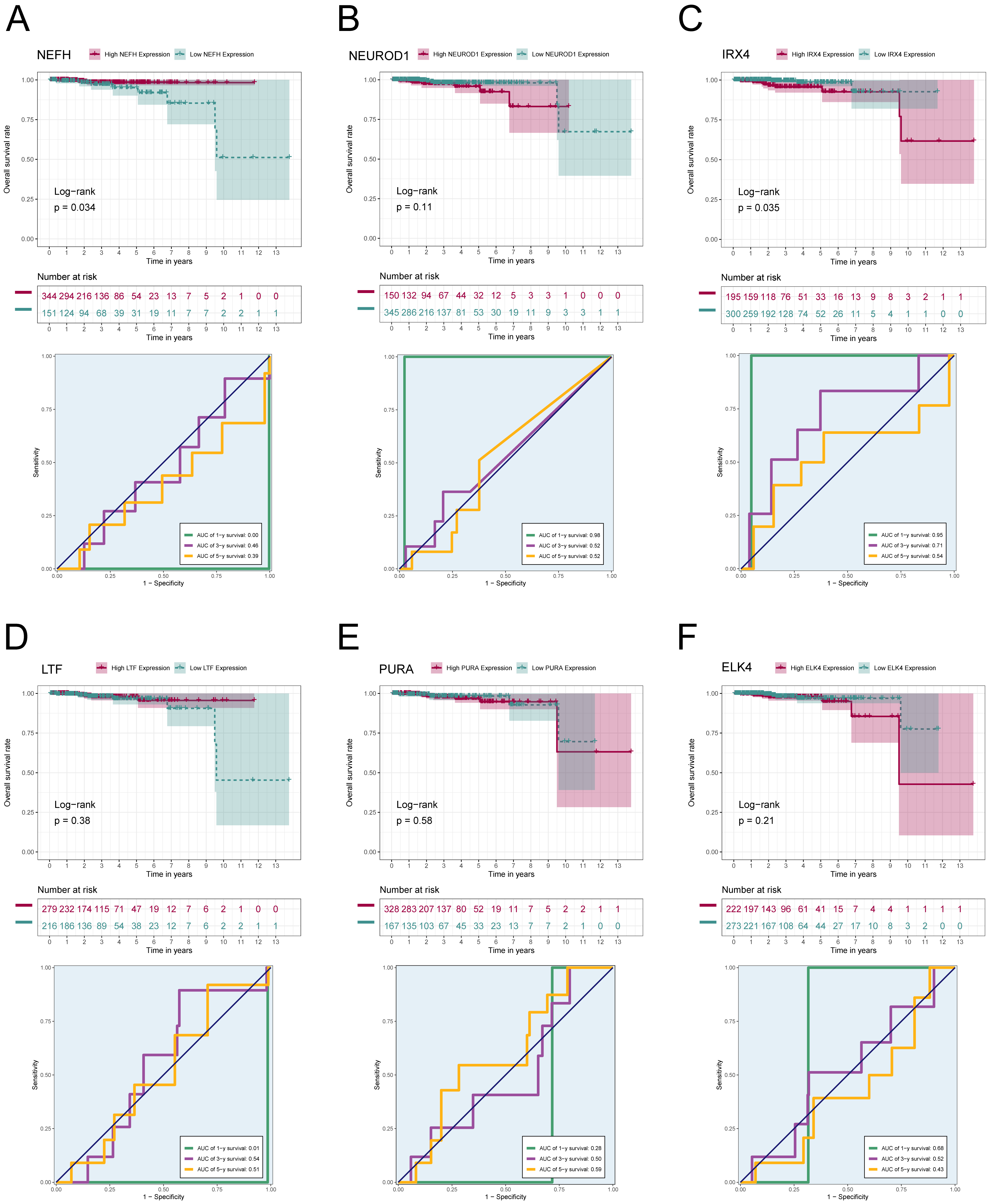

Supplement: Supplementary Figure 2 — The analysis of the C3 subtype marker gene and the top five TFs in bulk. Kaplan-Meier survival curves and ROC curves depicted the marked genes of C3 malignant cell subtype and the top five TFs (NEUROD1, IRX4, LTF, PURA, ELK4). *P < 0.05, **P < 0.01, ***P < 0.001, and ****P < 0.0001. “ns” was used to say that there was no significant difference. [file Image2.tif]
